# Evaluation Methods for Inference-Time Retrieval-Augmented and Graph Retrieval-Augmented Large Language Models in Health Care: Scoping Review

**DOI:** 10.2196/90046

**Published:** 2026-08-03

**Authors:** Yuhan Zhao, Yiqun Miao, Rongrong Guo, Yuan Luo, Huiying Wang, Ying Wu

**Affiliations:** 1 School of Nursing, Capital Medical University, No. 10 Xitoutiao, Youanmenwai, Fengtai District, Beijing, 100069, China, 86 13910789837

**Keywords:** large language models, natural language processing, artificial intelligence, retrieval-augmented generation, GraphRAG, information storage and retrieval, scoping review, evaluation studies as topic, hallucination, clinical decision support systems

## Abstract

**Background:**

Inference-time retrieval augmentation is increasingly used to improve the traceability and verifiability of large language model (LLM) applications in health care. Evaluation practices for text-based retrieval-augmented generation (RAG) and graph-structured RAG (GraphRAG) systems remain heterogeneous, which limits comparison across studies and complicates judgments about clinical readiness.

**Objective:**

This review mapped evaluation methods for inference-time retrieval-augmented and graph-structured retrieval-augmented LLM systems in health care and characterized how evaluation constructs are defined, operationalized, and reported across system layers and evaluation-setting categories.

**Methods:**

We conducted a scoping review in accordance with PRISMA-ScR (Preferred Reporting Items for Systematic Reviews and Meta-Analyses extension for Scoping Reviews), with search reporting informed by PRISMA-S (PRISMA literature search extension). Searches were conducted through May 14, 2026, in PubMed (MEDLINE), Web of Science Core Collection, IEEE Xplore, ACM Digital Library, arXiv, and medRxiv, with backward and forward citation tracking of included studies. Eligible records described health care–relevant LLM systems using inference-time RAG and reported at least 1 evaluation component. Data were charted on study characteristics, system design, retrieval-layer evaluation, evidence linkage, safety-related and GraphRAG-specific evaluation, and selected reporting and governance characteristics. We also constructed an evidence-and-gap map cross-classifying evaluation-setting categories with key evaluation domains.

**Results:**

A total of 157 studies met the inclusion criteria. Clinical question answering was the most frequently represented application (89/157, 56.7%), followed by clinical decision support (70/157, 44.6%). Most evaluations were conducted in offline-only settings (140/157, 89.2%), whereas 17/157 (10.8%) studies reported workflow-facing, prospective, or deployment-level evaluation. Independent retrieval-layer evaluation was reported in 47/157 (29.9%) studies. Grounding and faithfulness evaluation was reported in 41/157 (26.1%) studies, and fine-grained evidence verification was reported in 22/157 (14%) studies. Human evaluation was reported in 94/157 (59.9%) studies, but interrater reliability was reported in 26/94 (27.7%) studies. LLM-as-judge evaluation was reported in 41/157 (26.1%) studies, with bias-control measures reported in 15/41 (36.6%) studies. Formal safety-related evaluation was reported in 45/157 (28.7%) studies. Among 27 (17.2%) GraphRAG studies, intermediate-artifact evaluation was reported in 11/27 (40.7%) studies, and graph construction evaluation was reported in 6/27 (22.2%) studies. The evidence-and-gap map showed limited coverage of fine-grained verification, contradiction handling, safety evaluation, LLM-as-judge safeguards, GraphRAG construction evaluation, and GraphRAG intermediate-artifact evaluation in workflow-facing, prospective, or deployment-level settings.

**Conclusions:**

Evaluation of health care RAG and GraphRAG systems has expanded rapidly, yet reporting and operational definitions remain inconsistent across evaluation layers. Current evidence remains concentrated in offline evaluation, with limited workflow-facing, prospective, or deployment-level assessment of retrieval quality, fine-grained evidence linkage, safety, LLM-as-judge safeguards, GraphRAG construction quality, and GraphRAG intermediate artifacts. This review maps these gaps across evaluation-setting categories and translates them into synthesis-informed evaluation considerations. These findings suggest that future evaluation may need to move beyond end-to-end benchmark performance toward more transparent, layer-specific, safety-oriented, and clinically contextualized assessment before workflow-facing implementation.

## Introduction

### Rationale

Large language models (LLMs) are increasingly explored in health care for tasks such as clinical decision support, patient education, documentation, administrative workflows, and broader biomedical use cases [1-6]. However, study designs, evaluation end points, and reporting practices remain heterogeneous, and deployment in high-stakes medical settings is constrained by the tendency of these models to produce factually incorrect, internally inconsistent, or insufficiently supported statements [7,8]. Such reliability limitations raise patient safety concerns and can undermine clinician trust in automated systems [9,10]. Improving factual accuracy and enabling verifiable outputs have therefore become central objectives for medical artificial intelligence (AI) research [11]. Accordingly, evaluation is not only a measure of technical performance but also a prerequisite for judging whether health care LLM systems are sufficiently transparent, safe, and interpretable before use in progressively more clinical or workflow-facing settings.

To mitigate factual unreliability, researchers have increasingly adopted inference-time retrieval-augmented generation (RAG) [12]. This paradigm retrieves evidence from external knowledge sources such as clinical guidelines, biomedical literature, institutional protocols, and electronic health records (EHRs) during the generation process. By conditioning generation on retrieved context, these systems aim to improve accuracy and support traceability of outputs to source evidence [13]. The approach includes text-based RAG and emerging graph-structured RAG (GraphRAG) [14]. Many retrieval-augmented systems retrieve text using dense vector similarity or hybrid sparse-dense retrieval, whereas graph-based approaches use graph structure to condition inference-time evidence retrieval or organization for generation [15,16]. Graph-based methods can also introduce unique intermediate artifacts such as retrieved paths, retrieved subgraphs, and graph community summaries, which create additional evaluation targets beyond end-to-end task performance. However, retrieval augmentation does not by itself ensure that retrieved evidence is relevant, that generated claims are faithfully supported by that evidence, that citation and source attributions are correct, or that outputs are clinically safe. RAG and GraphRAG systems therefore require evaluation methods that distinguish retrieval quality, evidence linkage, end-to-end output quality, safety-related behavior, and readiness for more clinically realistic settings.

Despite the rapid expansion of health care retrieval-augmented architectures, particularly since 2024, evaluation methodologies remain fragmented. While systematic and scoping reviews have mapped general LLM applications in clinical medicine, patient education, and broader health care settings [2-6], other reviews and guidance papers have focused more directly on testing, evaluation, and reporting of health care LLM applications [1,6,11,17]. General RAG and GraphRAG reviews have also summarized retrieval-augmented architectures, retriever-generator integration, robustness issues, graph-based indexing, graph-guided retrieval, graph-enhanced generation, and emerging evaluation frameworks [12,15,16,18-20]. These syntheses are important, but they have not primarily been designed to provide a layer-specific synthesis of health care evaluation methods for inference-time RAG and GraphRAG systems, including verification granularity, independent retrieval-layer evaluation, formal safety-related evaluation, and evaluation-setting context. Many studies emphasize end-to-end performance metrics, which can obscure the distinct contributions and failure modes of retrieval and generation components [18,19].

RAG evaluation literature has further highlighted that retrieval-augmented systems pose distinctive evaluation challenges because their behavior depends on both retrieval and generation components as well as on dynamic external knowledge sources [18,19]. A related review of medical RAG literature likewise suggests that research in this area has concentrated on technical implementations and clinical applications, whereas evaluation commonly relies on automated metrics or broad human judgments, with less explicit attention to bias and safety [21]. In addition, it is often unclear to what extent evaluation protocols incorporate clinical validity, safety assessment, and testing in settings that approximate real clinical use [17,22,23]. Evaluation methods for inference-time RAG and GraphRAG systems therefore warrant dedicated synthesis, particularly to clarify how layer-specific constructs are operationalized, how evaluation coverage varies across evaluation-setting categories and key evaluation domains, and where methodological gaps remain within health care domains. To our knowledge, no previous review has specifically mapped how evaluation methods for health care inference-time RAG and GraphRAG systems are operationalized across retrieval-layer evaluation, grounding and faithfulness evaluation, citation and source correctness evaluation, formal safety-related evaluation, human evaluation, automated metrics, LLM-as-judge evaluation, GraphRAG construction and intermediate-artifact evaluation, and evaluation-setting categories.

### Objectives

Given the heterogeneity in task definitions, data sources, and evaluation end points, we conducted a scoping review to map the evidence and characterize evaluation practices rather than to estimate pooled effects. The primary objective of this review was to systematically map evaluation methods used for inference-time RAG and GraphRAG systems in health care. Specifically, this review aimed to characterize evaluation constructs, measurement approaches, and study designs across system layers, including retrieval-layer evaluation, grounding and faithfulness evaluation, citation and source correctness evaluation, verification granularity, end-to-end task outcomes, human evaluation, automated metrics, LLM-as-judge evaluation and related safeguards, formal safety-related evaluation, GraphRAG construction evaluation, and GraphRAG-specific intermediate-artifact evaluation. By synthesizing these practices into a layer-specific taxonomy and mapping evaluation coverage across evaluation-setting categories and key evaluation domains, this review aimed to identify methodological gaps, differentiate the evaluation needs of text-based RAG and GraphRAG systems, and inform more transparent, setting-appropriate, and safety-oriented evaluation and reporting in future research.

## Methods

### Protocol and Registration

This scoping review was designed to systematically map and characterize evaluation methods used in health care LLM systems using inference-time RAG, with specific attention to both text-based RAG and GraphRAG approaches. The review was conducted in accordance with methodological guidance from the Joanna Briggs Institute for scoping reviews and reported in accordance with the PRISMA-ScR (Preferred Reporting Items for Systematic Reviews and Meta-Analyses Extension for Scoping Reviews) checklist (Checklist 1) [24,25]. A protocol specifying the research questions, eligibility criteria, information sources, search strategy structure, screening procedures, data charting items, and coding framework was developed a priori and registered on the Open Science Framework (OSF) through OSF Registries before study selection and data charting were initiated (registration ID: MTF5X).

The objective was to identify and synthesize how evaluation constructs are defined and operationalized across system layers, to summarize study designs and evaluation modalities, to map evaluation coverage across descriptive evaluation-setting categories, and to characterize reporting practices relevant to the interpretation of evaluation methods, including governance indicators related to real patient data, deidentification, and ethics approval, exemption, or waiver. Specifically, this review sought to map how evaluation was performed at the retrieval-layer, grounding and faithfulness, evidence-verification, safety-related, GraphRAG-specific, and end-to-end system levels, how evaluation coverage varied across evaluation-setting categories, and how these evaluations were reported across heterogeneous health care settings. Given the expected heterogeneity in evaluation targets, end points, and study settings, a scoping review approach was selected to support comprehensive mapping and structured evidence synthesis rather than quantitative effect estimation.

This scoping review addressed 5 research questions aligned with the structure of the Results section.

First, what health care tasks and evaluation-setting categories are represented in studies evaluating inference-time RAG and GraphRAG LLM systems?

Second, what system design characteristics relevant to evaluation are reported, including knowledge source types, retrieval approaches, reranking stages, GraphRAG-related system features, and generator or evaluator model choices?

Third, how is retrieval-layer evaluation operationalized, including whether independent component-level evaluation is performed, which retrieval metrics are reported, how relevance labels or reference evidence are constructed, and how retrieval unit and granularity are specified?

Fourth, how are grounding and faithfulness outcomes evaluated, including how these constructs are operationalized, how outputs are linked to retrieved evidence, what units of analysis are used for verification, and whether citation and source correctness evaluation or conflict and contradiction handling is assessed?

Finally, what broader evaluation modalities and reporting practices are used, including human evaluation procedures, automated end point selection, LLM-as-judge evaluation procedures, formal safety-related evaluation, GraphRAG construction evaluation, GraphRAG intermediate-artifact evaluation, and selected governance-related elements?

### Ethical Considerations

Ethical approval was not required for this scoping review because it synthesized data from publicly available studies and did not involve human participants.

### Eligibility Criteria

Eligibility criteria were defined a priori using the Population, Concept, and Context (PCC) framework recommended for scoping reviews [25]. In this review, the population corresponded to implemented health care–relevant LLM systems, the concept to inference-time RAG and its evaluation, and the context to health care application settings. Records were eligible if they described a health care–relevant system in which an LLM produced generative outputs and implemented RAG by retrieving external evidence during inference and incorporating retrieved material into generation, including text-based retrieval as well as GraphRAG approaches in which graph structure was used for inference-time evidence retrieval, organization, or assembly. Records were required to report at least 1 evaluation component (eg, retrieval performance, grounding and faithfulness, citation and source correctness, end-to-end task performance, human evaluation, formal safety-related evaluation, or GraphRAG-specific evaluation).

We excluded studies describing training-only knowledge injection without inference-time retrieval conditioning, retrieval systems without generative outputs, studies without empirical evaluation of an implemented system, narrative reviews, conference abstracts without accessible full text, and records for which full text could not be obtained. When multiple records described the same underlying study, a single record was retained for synthesis to avoid double counting. We preferentially retained the peer-reviewed version when it adequately described the evaluation methods; otherwise, the record providing the most complete description of the study design, system implementation, and evaluation procedures was retained as the primary synthesis record, with companion reports consulted as needed for clarification only.

### Information Sources

We searched PubMed (MEDLINE), Web of Science Core Collection, IEEE Xplore, and the ACM Digital Library. Searches were conducted on May 14, 2026, and were limited to records published or posted from January 1, 2024 to May 14, 2026. Searches were limited to English-language records. This period was selected to focus on contemporary evaluation practices from 2024 onward, alongside the broader uptake of frontier LLMs and specialized health care RAG frameworks. This window was intended to reflect recent evaluation paradigms for inference-time retrieval architectures in clinical domains rather than foundational natural language processing (NLP) tasks that predated their widespread health care use. To capture emerging work disseminated ahead of journal publication, we additionally searched arXiv and medRxiv using equivalent concept blocks adapted to platform-specific syntax and the same search cutoff. We also performed backward and forward citation tracking for all included studies using the same eligibility criteria to identify additional eligible records [26].

### Search Strategy

The search strategy was developed iteratively by the review team and combined controlled vocabulary and free-text terms for (1) LLMs; (2) inference-time RAG, including GraphRAG approaches; (3) health care context; and (4) evaluation-related concepts. Controlled vocabulary was used where supported by the database, and syntax, fields, and limits were adapted to each platform. To align the search strategy with the review objective of synthesizing evaluation practices, the database queries included evaluation-related terms, including evaluation, benchmarking, metrics, grounding and faithfulness, hallucination, safety-related evaluation, and citation and source correctness, to improve identification of studies that explicitly reported evaluation methods, benchmarking designs, or evidence-verification procedures. Because the review focused specifically on evaluation practices, these terms were included to improve precision for studies reporting explicit evaluation methods; backward and forward citation tracking was used to mitigate the risk of missing eligible studies whose evaluation components were not captured in searchable titles, abstracts, or keywords. Literature searching and search reporting were conducted and documented in accordance with the PRISMA-S (PRISMA literature search extension) [26]; an item-by-item PRISMA-S checklist, full database-specific search strategies, and supplementary search procedures are provided in Checklist 2 and Multimedia Appendix 1. No study registries, structured website handsearching, contact-based supplementary identification, or formal search peer review was undertaken; these PRISMA-S items are reported explicitly in Checklist 2.

### Selection of Sources of Evidence

Retrieved records were deduplicated in EndNote (Clarivate) and screened in a dedicated platform. Moreover, 2 reviewers (YZ and YM) independently screened titles and abstracts and then full texts of records classified as potentially eligible or uncertain. Reviewers completed a calibration exercise before full screening. Discrepancies were resolved through a consensus-seeking discussion between the 2 reviewers (YZ and YM); if consensus could not be reached, a third senior reviewer (YW) adjudicated the final inclusion decision. Reasons for exclusion at the full-text stage were recorded and are reported in the PRISMA (Preferred Reporting Items for Systematic Reviews and Meta-Analyses) flow diagram. Records identified through all information sources and citation tracking were screened using the same eligibility criteria and selection procedures.

### Data Charting Process

A standardized data-charting form was developed a priori to operationalize the review questions and support consistent charting of system characteristics, evaluation constructs, operational definitions, descriptive evaluation-setting categories, evaluation domains, and reporting elements. The form was refined iteratively through team discussion and was piloted on a prespecified sample of included studies to calibrate interpretation of fields and coding rules. Following calibration, one reviewer (YZ) charted data from all included studies and a second reviewer (YM) independently verified all entries against the full texts and available supplementary materials. Discrepancies were resolved through a consensus-seeking discussion. If consensus was not reached, a third senior reviewer (YW) adjudicated the final coding to maintain consistency across the review.

Charting was conducted at the study level and allowed multilabel coding when studies reported multiple tasks, knowledge sources, evaluation-setting categories, or evaluation modalities. Coding was based on full-text review and prespecified category definitions rather than title, abstract, or terminology alone. When information relevant to a field was explicitly reported, fields were coded according to the relevant category definitions. When information was not reported, fields were coded as “not reported.” Fields were coded as “not applicable” when the item was structurally irrelevant to the study design or evaluation approach. Ambiguous cases were resolved through full-text review and reviewer consensus rather than retained as a separate coding category. Coding decisions and adjudication notes were documented to preserve an audit trail.

### Data Items

Data were charted across five domains: (1) study characteristics and application settings, including application task categories and descriptive evaluation-setting categories; (2) system design characteristics relevant to evaluation, including knowledge source type, retrieval granularity, retrieval approach, reranking, generation model choice, evaluator model choice, and GraphRAG-related system features; (3) retrieval-layer evaluation, including retrieval metrics, relevance labeling procedures, reference evidence construction, and reporting of document-level or passage-level granularity; (4) grounding and faithfulness evaluation, including terminology used, operationalization approach, unit of analysis, fine-grained evidence verification, citation and source correctness, and handling of conflicting or contradictory evidence; and (5) evaluation modalities and reporting-related study characteristics, including human evaluation design and rater characteristics, interrater reliability (IRR) reporting, automated metrics, LLM-as-judge procedures and bias-control measures, formal safety-related evaluation, GraphRAG construction and intermediate-artifact evaluation, and governance indicators when explicitly described.

### Operational Definitions and Coding Framework

To support consistent and reproducible synthesis, we applied an explicit coding framework with operational definitions for evaluation layers, constructs, and study attributes. The framework was developed before full data charting, refined during calibration, and then applied uniformly across all included studies. Key operational definitions and primary coding units used in this review are summarized in Table 1.

**Table 1.** Operational definitions and coding units used in this review.

Construct	Working definition in this review	Primary unit of assessment	Included under this construct	Not counted as this construct
Retrieval-layer evaluation	Explicit assessment of the quality or performance of retrieved evidence independent of final generated outputs.	Retrieved item, ranked set, document, passage, node, path, or subgraph	Retrieval metrics, relevance judgments, structured assessment of retrieval quality, comparison of retrieved evidence sets	End-to-end task performance reported without explicit retrieval-layer assessment
Grounding and faithfulness evaluation	Assessment of whether generated answers, claims, statements, citations, or sources were supported by retrieved evidence or retrieved context, and whether generated outputs remained constrained by that evidence.	Response, answer, claim, statement, citation, source, or sentence	Evidence-support assessment, answer faithfulness assessment, source-supported output checking, claim verification against retrieved evidence, and assessment of unsupported additions relative to retrieved context	Factual correctness assessed only against an external reference standard without explicit linkage to retrieved evidence
Citation and source correctness evaluation	Assessment of whether cited or displayed sources existed, were accurate, and supported the corresponding answer content.	Citation, source, source excerpt, or linked answer segment	Source attribution checks, citation support checks, source existence checks, evidence-to-answer linkage assessment	Citation presence alone without verification that the cited or displayed source supports the answer content
Formal safety-related evaluation	Assessment in which safety, harm, unsafe recommendations, hallucination-related risk, undertriage, suicide risk, harmful content, or comparable safety outcomes were included as formal evaluation dimensions or end points.	Response, recommendation, decision, triage output, risk classification, or system behavior	Safety scores, harmfulness ratings, unsafe recommendation assessment, hallucination risk assessment, undertriage or overtriage harm evaluation, harmful content blocking	General discussion of safety risks without formal evaluation; accuracy or guideline concordance reported without a safety-related end point
Evaluation-setting category	Descriptive classification of evaluation conditions by their relationship to real-world health care use, rather than an ordinal maturity level.	Study or evaluation setting	Offline-only evaluation, simulated vignette or case evaluation, workflow pilot or user study, prospective clinical study, real-world deployment or postdeployment monitoring	General study setting description without enough information to classify the evaluation setting
GraphRAG^a^ minimum criterion	Explicit use of graph structure at inference time to retrieve, organize, or assemble evidence that conditions generation.	System-level design feature	Retrieval of nodes, paths, subgraphs, graph-structured evidence assembly, graph-derived summaries, or graph-derived community summaries used at inference time	Knowledge graph use limited to background knowledge representation, training-time enrichment, or architecture description without graph-structured inference-time retrieval or evidence assembly
Graph construction evaluation	Explicit evaluation of graph construction quality or graph content quality.	Graph, node, edge, relation, triple, or graph-derived schema	Assessment of node correctness, edge correctness, relation quality, triple extraction quality, graph completeness, or graph construction accuracy	Reporting graph size, graph architecture, or graph construction workflow without evaluating graph quality
GraphRAG intermediate-artifact evaluation	Explicit evaluation of intermediate graph-related artifacts produced or used during graph-structured retrieval-augmented generation.	Node, edge, path, subgraph, graph-derived summary, retrieved graph context, or provenance-linked graph artifact	Assessment of retrieved nodes, retrieved paths, retrieved subgraphs, graph-derived summaries, graph-based context, or provenance-linked graph artifacts	End-to-end output evaluation of a GraphRAG system without assessment of graph-related intermediate artifacts

a GraphRAG: graph-structured retrieval-augmented generation.

Evaluation targets were conceptualized as nonmutually exclusive layers reflecting the multicomponent nature of inference-time RAG systems, including retrieval-layer evaluation, grounding and faithfulness evaluation, and end-to-end task evaluation. In addition, studies were coded for cross-cutting evaluation modalities and reporting dimensions, including human evaluation, formal safety-related evaluation, efficiency and implementation-readiness indicators, graph construction evaluation, GraphRAG intermediate-artifact evaluation, and reporting and governance dimensions.

Retrieval-layer evaluation was coded as present only when a study reported an explicit assessment of retrieved evidence quality independent of final generated outputs. This included quantitative retrieval metrics, relevance judgments of retrieved items, or structured evaluation of retrieval performance. Studies that reported only end-to-end task performance or qualitative examples without explicit assessment of retrieved evidence were not coded as reporting retrieval-layer evaluation.

For the purposes of this review, grounding and faithfulness evaluation was defined as assessment of whether generated answers, claims, statements, citations, or sources were supported by retrieved evidence or retrieved context, and whether generated outputs remained constrained by that evidence. Because terminology varied across studies, grounding and faithfulness evaluations were identified based on described verification procedures rather than author-reported labels. Citation and source correctness, claim verification against retrieved evidence, and assessment of unsupported additions relative to retrieved context were included when they explicitly evaluated alignment between generated outputs and retrieved sources. Hallucination-related assessments were included under grounding and faithfulness only when unsupported content was evaluated with reference to retrieved evidence or retrieved context. This operationalization was informed by previous RAG evaluation literature that assesses answer faithfulness and related evidence-linked dimensions [18,20].

For each distinct grounding or evidence-linkage evaluation component, we recorded the most granular verification unit explicitly described, including response- or answer-level, claim- or statement-level, citation- or source-level, and sentence-level verification. Because individual studies could report multiple evaluation components at different verification units, the summary granularity categories were nonmutually exclusive. Fine-grained evidence verification was coded when studies reported claim-, statement-, citation-, source-, or sentence-level verification. When a relevant verification unit was not explicitly described, it was coded as “not reported.” Fields were coded as “not applicable” when the item was structurally irrelevant to the study design or evaluation approach. Ambiguous cases were resolved through full-text review and reviewer consensus rather than retained as a separate coding category.

Evaluation-setting categories were coded to describe the relationship between evaluation conditions and real-world health care use [22]. These descriptive, nonmutually exclusive categories included offline-only evaluation, simulated vignette or case evaluation, workflow pilot or user study, prospective clinical study, and real-world deployment or postdeployment monitoring. These categories were not treated as ordinal maturity levels. When a study reported multiple evaluation-setting categories, all applicable categories were recorded.

GraphRAG systems were identified using a minimum criterion requiring explicit use of graph structure at inference time to retrieve, organize, or assemble evidence that conditioned generation [27]. This included retrieval of nodes, paths, subgraphs, graph-derived summaries, or graph-derived community summaries. Studies that referenced the use of a knowledge graph without inference-time graph-structured retrieval or evidence assembly were not classified as GraphRAG. Classification was based on the reported inference-time role of graph structure in evidence retrieval or assembly rather than on the mere presence of a knowledge graph within the broader system architecture. Graph construction evaluation was coded separately when studies evaluated graph nodes, edges, relations, triples, or knowledge-graph construction quality. GraphRAG intermediate-artifact evaluation was coded when studies evaluated retrieved nodes, retrieved paths, retrieved subgraphs, graph-derived summaries, graph-based context, or provenance-linked graph artifacts.

When a study reported multiple tasks, multiple knowledge sources, or multiple evaluation methods, all applicable categories were coded. Information relevant to a field but not explicitly reported was coded as “not reported.” Items that were structurally irrelevant to a study design or evaluation approach were coded as “not applicable.” Coding decisions and adjudication notes were documented to preserve traceability [11].

### Critical Appraisal of Individual Sources of Evidence

Consistent with scoping review methodology, we did not conduct formal methodological quality appraisal or risk-of-bias assessment [24,25]. This was because the aim of the review was to map the range and characteristics of evaluation practices rather than to estimate intervention effects or exclude studies on the basis of methodological quality.

### Reporting-Related Assessment

We conducted a structured assessment of evaluation reporting completeness to characterize transparency of evaluation practices and to support interpretation of methodological gaps across the evidence base. This assessment was descriptive rather than evaluative and was intended to characterize reporting transparency, not to rate methodological quality. Reporting assessment focused on whether key information necessary to interpret and compare evaluation results was explicitly described [11].

For each included study, we recorded reporting of key system and evaluation elements, including (1) retrieval and knowledge source description, including corpus or source, retrieval unit and granularity, retriever, and reranking components; (2) grounding and faithfulness evaluation, including definition, operationalization, and unit of analysis; (3) human evaluation procedures, including rater background and IRR; (4) automated evaluation procedures, including automated metrics, LLM-as-judge evaluation, and bias-control measures specifically applied when an LLM was used as an evaluator; (5) formal safety-related evaluation; and (6) GraphRAG construction evaluation and GraphRAG intermediate-artifact evaluation when applicable.

For studies involving real patient data, we additionally recorded reporting of ethical governance elements, including deidentification procedures and institutional review board approval, exemption, or waiver [11,22]. No studies were excluded on the basis of reporting completeness or ethical reporting. Findings were synthesized descriptively to identify common reporting gaps and areas where standardized reporting guidance could improve transparency and comparability in future studies.

### Synthesis of Results

Given the heterogeneity in health care tasks, system architectures, evaluation targets, and outcome measures, quantitative meta-analysis was not appropriate. We therefore synthesized findings using descriptive statistics and narrative synthesis to map evaluation practices across included studies [24]. For the structured charting domains, we summarized counts and proportions of studies reporting the corresponding evaluation construct or design element.

Studies were retained as the unit of analysis, and multilabel coding was permitted when a study reported multiple tasks, evaluation layers, knowledge sources, or evaluation-setting categories. Results were organized to reflect the layer-specific evaluation framework defined a priori. Synthesis addressed application settings and evaluation contexts; system design patterns relevant to evaluation; retrieval-layer evaluation; grounding and faithfulness evaluation; evaluation modalities, including human evaluation and LLM-as-judge evaluation; formal safety-related evaluation; GraphRAG construction and intermediate-artifact evaluation; descriptive evaluation-setting categories; and selected reporting and governance observations.

Where informative, cross-tabulations were used to support descriptive comparisons across task categories, evaluation-setting categories, evaluation domains, and system design features; no statistical hypothesis testing was performed. Evaluation-setting categories were used descriptively and were not analyzed as an ordinal maturity scale. Findings were summarized using tables and figures to support transparency and interpretability. Each included study contributed equally to the synthesis.

To visualize evaluation gaps, we constructed an evidence-and-gap map by cross-classifying descriptive evaluation-setting categories with key evaluation domains. Evaluation-setting categories included offline-only evaluation, simulated vignette or case evaluation, workflow pilot or user study, prospective clinical study, and real-world deployment or postdeployment monitoring. Evaluation domains included retrieval-layer evaluation, fine-grained evidence verification, citation and source correctness evaluation, conflict or contradiction handling, formal safety-related evaluation, human evaluation with IRR reporting, LLM-as-judge evaluation with bias-control measures, GraphRAG construction evaluation, and GraphRAG intermediate-artifact evaluation. Evaluation-setting categories and evaluation domains were coded as nonmutually exclusive; therefore, individual studies could contribute to more than one cell.

## Results

### Search Results and Study Selection

**Figure 1.**
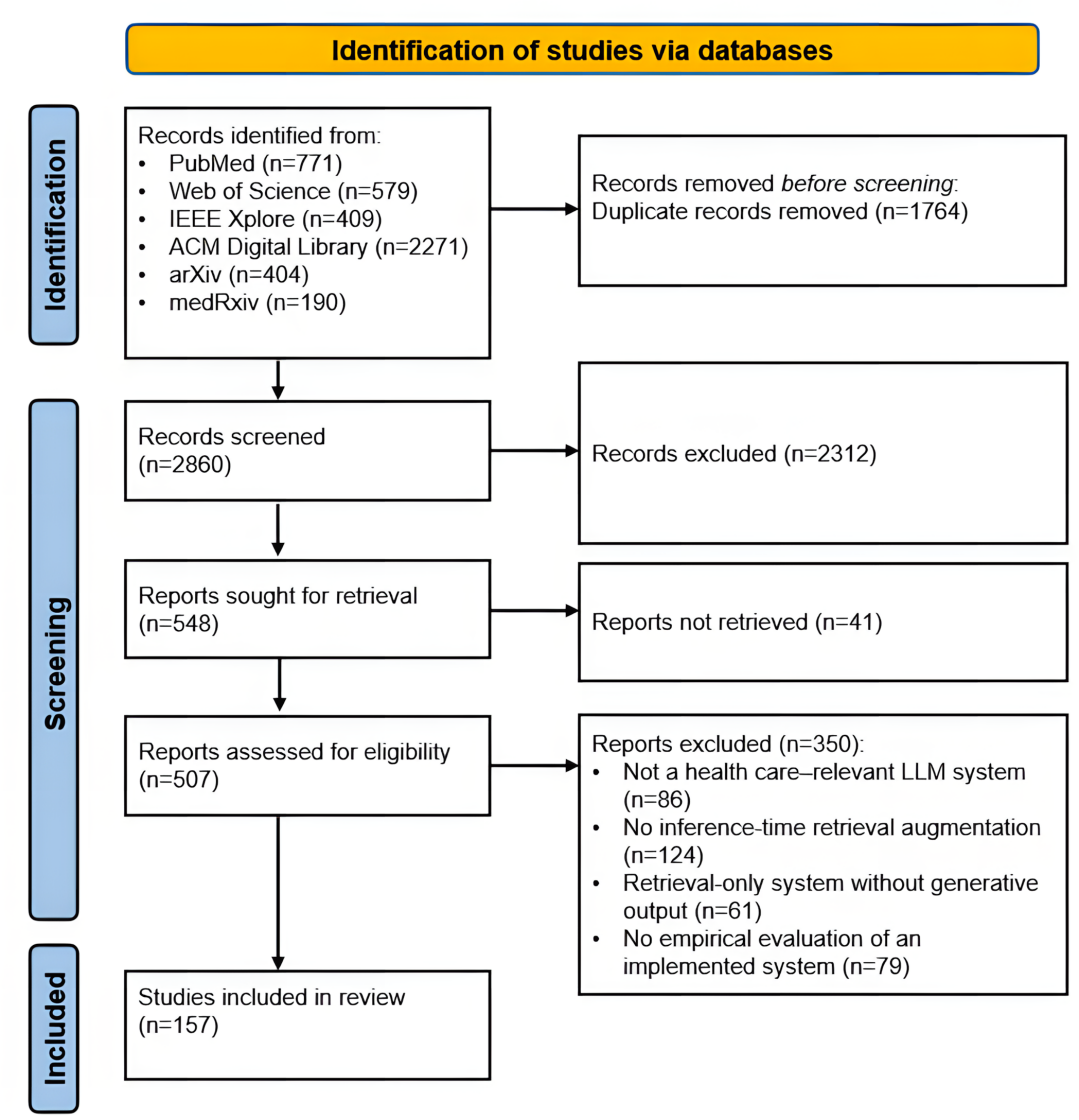
PRISMA-ScR (Preferred Reporting Items for Systematic Reviews and Meta-Analyses extension for Scoping Reviews) flow diagram of the study selection process. This diagram outlines the systematic literature search and screening procedure conducted in accordance with PRISMA-ScR guidelines. It details the number of records identified from databases and preprint platforms (PubMed, Web of Science Core Collection, IEEE Xplore, ACM Digital Library, arXiv, and medRxiv), the number of duplicates removed, and the stepwise exclusion reasons applied during screening and full-text eligibility assessment. A final total of 157 studies met the inclusion criteria for the scoping review. Searches were conducted through May 14, 2026. Backward and forward citation tracking was performed for all included studies. LLM: large language model.

A total of 157 studies met the inclusion criteria and were included in this scoping review [28-184]. The process of study identification, screening, and inclusion is summarized in the PRISMA-ScR flow diagram (Figure 1).

### Study Characteristics and Application Settings

The included studies (N=157 [28-184]) covered a broad range of health care tasks and evaluation contexts (Table 2; Table S1 in Multimedia Appendix 2). Clinical question answering was the most frequently studied application, reported in 89 (56.7%) studies. Other commonly reported applications included clinical decision support tasks (n=70, 44.6%), patient or caregiver education (n=27, 17.2%), and summary or report generation (n=24, 15.3%). Task categories were not mutually exclusive, and some systems addressed multiple downstream tasks. Overall, the evidence base remained concentrated in question answering and decision-support applications, with smaller clusters of patient education, report generation, medical visual question answering, and administrative or operational support applications.

**Table 2.** Characteristics of included studies and application settings (N=157). Percentages use all included studies (N=157) as the denominator. Application task categories, evaluation-setting categories, and knowledge-source categories were coded as multilabel categories; percentages therefore are not expected to sum to 100% within those blocks. Offline-only evaluation indicates the absence of workflow-facing, prospective, or deployment-level evaluation; studies could also be coded as simulated vignette or case evaluation when applicable. Evaluation-setting categories were descriptive, nonmutually exclusive categories rather than ordinal maturity levels. Full study-level coding is provided in Table S1 in Multimedia Appendix 2.

Domain and item	Count, n (%)
Application task categories	
Clinical question answering	89 (56.7)
Clinical decision support	70 (44.6)
Patient or caregiver education	27 (17.2)
Summary or report generation	24 (15.3)
Medical visual question answering	7 (4.5)
Medical imaging	6 (3.8)
Evaluation settings	
Offline-only evaluation	140 (89.2)
Simulated vignette or case evaluation	37 (23.6)
Any workflow-facing, prospective, or deployment-level evaluation	17 (10.8)
Workflow pilot or user study	17 (10.8)
Prospective clinical study	2 (1.3)
Real-world deployment or postdeployment monitoring	3 (1.9)
Knowledge source categories	
Public or public-mixed knowledge sources	119 (75.8)
Private institutional knowledge sources, including mixed sources	27 (17.2)
Graph-structured knowledge sources	7 (4.5)
EHR^a^-related subset	
Retrieval corpus explicitly EHR-related	21 (13.4)

a EHR: electronic health record.

**Figure 2.**
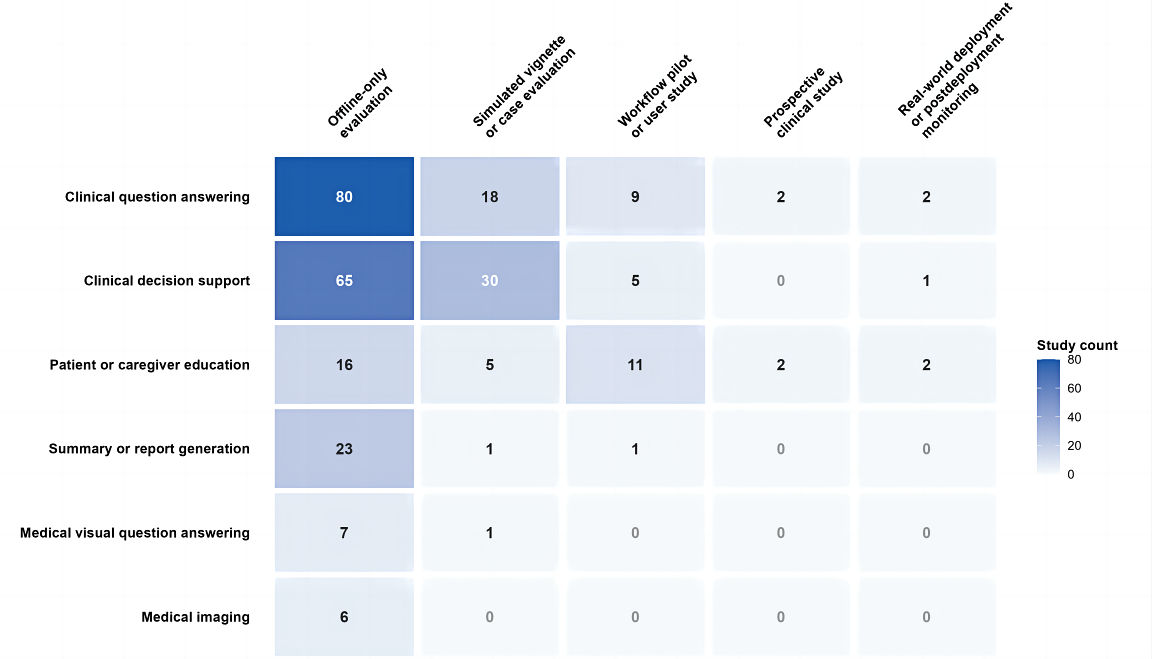
Heat map of health care application tasks by evaluation-setting category. The heat map shows study counts across health care application tasks and evaluation-setting categories, with evaluation-setting categories ordered descriptively from offline-only evaluation to real-world deployment or postdeployment monitoring (offline-only evaluation, simulated vignette or case evaluation, workflow pilot or user study, prospective clinical study, and real-world deployment or postdeployment monitoring). Cell labels indicate the number of included studies in each task-setting combination. Because application task and evaluation-setting category were coded as nonmutually exclusive categories, individual studies could contribute to multiple cells; therefore, row and column totals do not sum to the 157-study corpus. Evaluation-setting categories were used descriptively and were not treated as ordinal maturity levels. Full study-level coding is provided in Table S1 in Multimedia Appendix 2.

Across evaluation-setting categories, 140 (89.2%) studies reported offline-only evaluation and did not describe workflow pilots, prospective studies, or real-world deployment or postdeployment monitoring (Table 2; Table S1 in Multimedia Appendix 2). Simulated vignette or case evaluation was reported in 37 (23.6%) studies. Only 17 (10.8%) studies reported at least 1 workflow-facing, prospective, or deployment-level evaluation setting. This included workflow pilot or user study (n=17, 10.8%), prospective clinical study (n=2, 1.3%), and real-world deployment or postdeployment monitoring (n=3, 1.9%). These evaluation-setting categories were descriptive and not mutually exclusive. Figure 2 visualizes the relationship between application task category and evaluation-setting category.

Knowledge sources used for retrieval varied across studies (Table 2; Table S1 in Multimedia Appendix 2). Public or public-mixed knowledge sources were reported in 119 (75.8%) studies, whereas 27 (17.2%) studies used private institutional sources either exclusively or in combination with other sources. Graph-structured knowledge sources were coded as the primary knowledge-source category in 7 (4.5%) studies. Retrieval corpora explicitly described as EHR-related were reported in 21 (13.4%) studies.

### RAG System Design Patterns Relevant to Evaluation

System architectures and retrieval configurations were heterogeneous. Dense or vector-based retrieval was commonly reported, often alongside hybrid sparse-dense retrieval, reranking, or domain-specific corpus construction. Systems meeting the prespecified GraphRAG minimum criterion were reported in 27 (17.2%) studies. This count reflects studies in which graph structure participated in inference-time retrieval, evidence organization, reasoning, or generation, rather than studies that merely referenced a knowledge graph as background knowledge.

### Evaluation-Method Coverage Across Included Studies

Evaluation-method coverage across the included studies is summarized in Table 3. Overall, end-to-end task performance evaluation was reported more consistently than layer-specific retrieval assessment, evidence-linkage verification, citation and source correctness evaluation, conflict or contradiction handling, GraphRAG-specific artifact evaluation, and implementation-facing evaluation indicators.

Table 3 provides the corresponding counts and percentages for the principal evaluation domains, GraphRAG indicators, and governance-related reporting elements.

**Table 3.** Evaluation methods, graph-structured retrieval-augmented generation (GraphRAG) indicators, and governance-related reporting elements. Unless otherwise specified, percentages use all included studies (N=157) as the denominator. Interrater reliability uses the subgroup denominator of studies with human evaluation (n=94). Bias-control measures among large language model (LLM)–as-judge studies use the subgroup denominator of studies using LLM-as-judge evaluation (n=41). GraphRAG intermediate-artifact evaluation and graph construction evaluation use the subgroup denominator of studies meeting the GraphRAG minimum criterion (n=27). Deidentification and institutional review board (IRB) reporting use the subgroup denominator of studies using real patient data (n=49). Fine-grained evidence verification includes claim-level, statement-level, citation-level, source-level, or sentence-level verification. Evidence-verification granularity categories were nonmutually exclusive because individual studies could report distinct evaluation components at different verification units. Data are aligned with Tables S2 and S3 in Multimedia Appendix 2.

Domain and item	Studies, n/N (%)
Retrieval-layer evaluation	
Explicit retrieval-layer evaluation	47/157 (29.9)
Grounding and evidence linkage	
Grounding and faithfulness evaluation	41/157 (26.1)
Fine-grained evidence verification	22/157 (14)
Citation and source correctness evaluation	12/157 (7.6)
Conflict or contradiction handling evaluation	9/157 (5.7)
Evidence-verification granularity	
No evidence-support verification	116/157 (73.9)
Answer-level verification	20/157 (12.7)
Claim-level or statement-level verification	17/157 (10.8)
Citation-level or source-level verification	21/157 (13.4)
Evaluation modalities	
Human evaluation used	94/157 (59.9)
Interrater reliability reported among studies with human evaluation	26/94 (27.7)
Automated metrics reported	117/157 (74.5)
LLM-as-judge evaluation used	41/157 (26.1)
	
Bias-control measures reported among studies using LLM-as-judge evaluation	15/41 (36.6)
Safety-related evaluation	
Formal safety-related evaluation	45/157 (28.7)
GraphRAG-specific indicators	
Studies meeting GraphRAG minimum criterion	27/157 (17.2)
GraphRAG intermediate-artifact evaluation reported	11/27 (40.7)
Graph construction evaluation reported	6/27 (22.2)
Governance among studies using real patient data	
Studies using real patient data	49/157 (31.2)
Deidentification reported among studies using real patient data	33/49 (67.3)
IRB approval, exemption, or waiver reported among studies using real patient data	23/49 (46.9)

### Retrieval-Layer Evaluation

Independent retrieval-layer evaluation was reported in 47 (29.9%) studies (Table 3; Table S2 in Multimedia Appendix 2). In the remaining 110 (70.1%) studies, retrieval-layer evaluation was not reported, and evaluation was conducted at the level of final generated outputs or through qualitative examples. Thus, although all included systems used inference-time RAG, only a subset reported retrieval as a separable evaluation layer.

Among the 47 studies reporting retrieval-layer evaluation, commonly reported metric families included precision-based metrics (n=23, 48.9%), recall metrics (n=32, 68.1%), mean reciprocal rank (n=8, 17%), and normalized discounted cumulative gain (n=6, 12.8%; Table S2 in Multimedia Appendix 2). Reporting of retrieval-layer evaluation varied substantially across studies. Some studies reported standard information retrieval metrics, whereas others used context precision, context recall, retrieval accuracy, relevance judgments, or structured assessment of retrieved evidence. Studies that only compared final answer accuracy, final task performance, or qualitative examples without explicit assessment of retrieved evidence were not counted as reporting retrieval-layer evaluation.

### Evaluation of Grounding and Faithfulness

Grounding and faithfulness evaluation was reported in 41 (26.1%) studies (Table 3; Table S2 in Multimedia Appendix 2). Operationalizations varied across studies. Citation and source correctness evaluation was reported in 12 (7.6%) studies, and fine-grained evidence verification was reported in 22 (14%) studies. Conflict or contradiction handling evaluation was reported in 9 (5.7%) studies. These categories were not mutually exclusive.

Studies also varied in the granularity at which evidence linkage was assessed (Table 3; Table S2 in Multimedia Appendix 2). Answer-level verification was reported in 20 (12.7%) studies. Claim-level or statement-level verification was reported in 17 (10.8%) studies. Citation-level or source-level verification was reported in 21 (13.4%) studies. Overall, 116 of 157 studies (73.9%) reported no evidence-support verification. Verification units were therefore often broad, incompletely specified, or absent.

### Evaluation Modalities: Human and Automated End Points

Human evaluation was reported in 94 (59.9%) studies (Table 3; Table S3 in Multimedia Appendix 2). IRR was reported in 26 (27.7%) of the human-evaluated studies.

Automated metrics were reported in 117 (74.5%) studies (Table 3; Table S3 in Multimedia Appendix 2). These included task-performance metrics, retrieval metrics, lexical-overlap or semantic-similarity metrics, and automated evaluation frameworks, depending on the study design and evaluation target.

LLM-as-judge evaluation was reported in 41 (26.1%) studies. Among the 41 (26.1%) studies using LLM-as-judge evaluation, 15 (36.6%) studies reported at least 1 bias-control measure (Table 3; Table S3 in Multimedia Appendix 2).

Formal safety-related evaluation was reported in 45 (28.7%) studies. Safety was operationalized heterogeneously across studies, including explicit safety ratings, clinical risk or harm rubrics, medication and contraindication safety end points, harmful-content blocking, suicide risk stratification, triage harm indices, regulatory harm assessment, and hallucination-related risk evaluation. Table 4 summarizes the primary safety-related operationalization family assigned to each study with formal safety-related evaluation. Methodological safeguards, such as IRR reporting and bias-control measures for LLM-as-judge evaluation, were less frequently reported than the use of evaluation end points themselves.

**Table 4.** Primary operationalization families for formal safety-related evaluation among studies with formal safety-related assessment (n=45). Categories reflect the primary safety-related operationalization family assigned to each study with formal safety-related evaluation (n=45). Categories are mutually exclusive for this main-text summary, although individual studies could include secondary safety-related elements. Study-level coding is provided in Table S3 in Multimedia Appendix 2. Percentages use studies with formal safety-related evaluation (n=45) as the denominator.

Primary operationalization family	Studies, n (%)	Operationalization and evaluation modality
Explicit safety, harm, or clinical-risk scoring within expert or LLM^a^ evaluation rubrics	22 (48.9)	Broad safety scoring, harmfulness ratings, clinical-risk ratings, or safety dimensions embedded in expert, clinician, user, or LLM-as-judge evaluation rubrics.
Clinical management safety end points and harm consequences	10 (22.2)	Medication safety, drug contraindication screening, antibiotic or opioid safety, refusal or escalation behavior, undertriage or overtriage harm, medical or regulatory harm, or other task-specific clinical safety end points.
Hallucination, factuality, or evidence misalignment framed as safety	6 (13.3)	Hallucination, factuality, source suitability, unsupported clinical content, or evidence misalignment explicitly treated as a safety-relevant failure mode.
Harmful-content, crisis, adversarial, or misuse-safety safeguards	5 (11.1)	Harmful-content blocking, crisis safeguards, adversarial safety prompting, misuse-oriented testing, or safety monitoring for mental health or patient-facing systems.
Public-health misinformation and fact-checking safety evaluation	2 (4.4)	Fact-checking or misinformation evaluation framed as public-health risk mitigation.

a LLM: large language model.

### Exploratory Findings for GraphRAG-Specific Evaluation

In total, 27 studies met the prespecified minimum GraphRAG criterion and were included in GraphRAG-specific analyses (Table 3; Table S3 in Multimedia Appendix 2). All 27 GraphRAG studies reported end-to-end output evaluation. GraphRAG intermediate-artifact evaluation was reported in 11 of the 27 (40.7%) GraphRAG studies. Graph construction evaluation was reported in 6 (22.2%) studies. Evaluation of retrieved paths, subgraphs, graph-derived summaries, or provenance-linked graph artifacts remained uncommon. Given the still limited number of studies meeting the minimum GraphRAG criterion, these findings should be interpreted as exploratory.

### Reporting, Governance, and Study-Level Audit Trail

**Figure 3.**
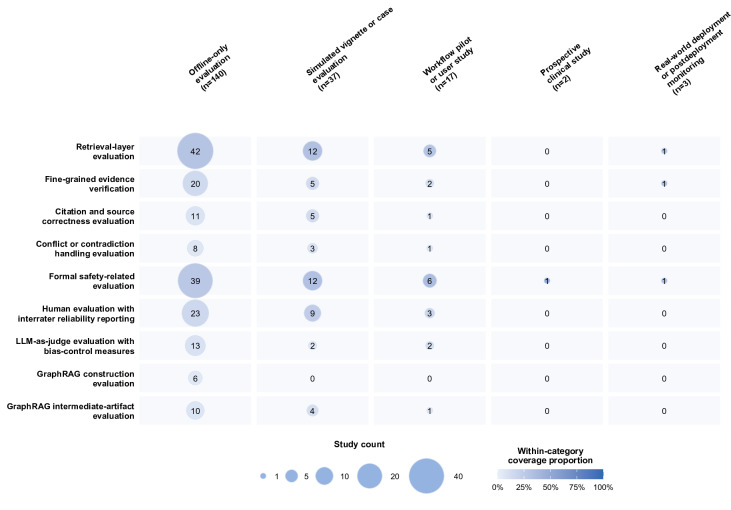
Evidence-and-gap map of evaluation domains by evaluation-setting category. The evidence-and-gap map cross-classifies descriptive evaluation-setting categories with key evaluation domains. Evaluation-setting categories include offline-only evaluation, simulated vignette or case evaluation, workflow pilot or user study, prospective clinical study, and real-world deployment or postdeployment monitoring. Evaluation domains include retrieval-layer evaluation, fine-grained evidence verification, citation and source correctness evaluation, conflict or contradiction handling, formal safety-related evaluation, human evaluation with interrater reliability reporting, LLM-as-judge evaluation with bias-control measures, GraphRAG construction evaluation, and GraphRAG intermediate-artifact evaluation. Circle area indicates the number of studies reporting the corresponding evaluation domain. Fill intensity indicates within-category coverage proportion, calculated as the number of studies in a given evaluation-setting category reporting the evaluation domain divided by the total number of studies in that category. Evaluation-setting categories and evaluation domains were coded as nonmutually exclusive; therefore, counts across rows or columns were not expected to sum to the total corpus. Source data for the map are provided in Table S4 in Multimedia Appendix 2. GraphRAG: graph-structured retrieval-augmented generation; LLM: large language model.

Reporting-related details relevant to the interpretation of evaluation procedures varied across the included studies. Graph-specific reporting and governance indicators also varied across studies (Table 3; Table S3 in Multimedia Appendix 2). Study-level reporting of knowledge-source type, indexing unit, and retriever type was heterogeneous, and graph-specific evaluation of intermediate artifacts was uncommon even among studies meeting the minimum GraphRAG criterion. Real patient data use was reported in 49 (31.2%) studies. Among these studies, deidentification procedures were reported in 33 (67.3%) studies and institutional review board approval, exemption, or waiver was reported in 23 (46.9%) studies. Concise study-level core coding tables and the source data for Figure 3 are provided in Tables S1-S4 in Multimedia Appendix 2.

### Evidence and Gap Map by Evaluation-Setting Category

Figure 3 presents an evidence-and-gap map (Table S4 in Multimedia Appendix 2) cross-classifying descriptive evaluation-setting categories with key evaluation domains. The map shows that evaluation coverage was concentrated in offline-only and simulated case-based evaluation categories. Across workflow-facing and prospective settings, coverage was sparse for fine-grained evidence verification, conflict or contradiction handling, formal safety-related evaluation, IRR reporting among studies with human evaluation, judge bias-control measures for LLM-as-judge evaluation, GraphRAG construction evaluation, and GraphRAG intermediate-artifact evaluation. This pattern indicates that the literature has developed more extensively for offline and simulated case-based evaluation than for workflow-facing, safety-oriented, governance-aware, or graph-artifact-specific evaluation.

### Synthesis-Informed Evaluation Considerations by Intended Evaluation Setting

Based on the mapped evaluation gaps, Table 5 presents synthesis-informed evaluation considerations by intended evaluation setting. This table is intended as a practice-oriented synthesis rather than an empirically validated checklist, implementation guideline, or ordinal maturity scale. Because the included studies were concentrated in offline-only and simulated evaluations, and because real-world deployment or postdeployment monitoring was sparsely represented, considerations for workflow-facing, prospective, and deployment-level settings should be interpreted as future-oriented evaluation considerations informed by the observed gaps and the implementation-relevant issues identified in this review.

**Table 5.** Synthesis-informed evaluation considerations by intended evaluation setting. This table is a practice-oriented synthesis based on the observed evaluation gaps and implementation-relevant considerations. It is not an empirically validated reporting checklist, implementation guideline, or ordinal maturity scale. The elements should be interpreted as considerations that may be adapted to task risk, user group, knowledge source, and intended use context.

Evaluation setting and use context	Evaluation components to consider	Evidence expectations and common failure modes
Offline-only evaluation: retrospective benchmark or held-out test set without workflow embedding	Retrieval-layer metrics; end-to-end task metrics; at least one evidence-linkage check at response or claim level; explicit reporting of corpus, retriever, and generator	Reference evidence or relevance labels; retrieval unit and granularity; prompts and configuration sufficient to interpret results. Common failures include retrieval miss, unsupported synthesis, and benchmark overfitting.
Simulated vignette or case evaluation: simulated clinician- or patient-facing scenarios	Offline elements plus scenario-based human review; claim- or citation-level verification when feasible; contradiction handling; at least one task-relevant safety-related end point	Expert-authored or expert-curated cases; adjudication rule or clinician rubric; explicit handling of conflicting evidence. Common failures include unsafe advice, weak abstention, and brittle behavior under conflicting evidence.
Workflow pilot or user study: interaction in a workflow-resembling environment with intended users	Simulated-setting elements plus usability and workflow outcomes; time burden or efficiency; user trust and calibration; escalation or handoff assessment	Clearly described user group; protocolized task flow; predefined safety oversight during pilot use. Common failures include workflow disruption, overtrust, hidden latency, and poor handoff to clinicians.
Prospective clinical study: prospective assessment in live or near-live care processes	Workflow-pilot elements plus predefined safety monitoring; subgroup analysis; governance documentation; protocolized human oversight	Prospective protocol; monitoring triggers; incident definitions; ethics and governance reporting. Common failures include consequential harm, performance heterogeneity, and inadequate monitoring thresholds.
Real-world deployment or postdeployment monitoring: operational deployment or postimplementation surveillance	Ongoing drift surveillance; incident logging; feedback loops; periodic re-audit of retrieval and evidence-linkage performance; version tracking	Monitoring cadence; trigger thresholds; rollback or escalation pathways; clear governance ownership. Common failures include performance drift, silent failure, configuration drift, and surveillance without remediation.

## Discussion

### Principal Findings

This scoping review mapped how inference-time RAG and GraphRAG systems in health care were evaluated across retrieval, evidence linkage, end-to-end performance, safety, reporting, and evaluation-setting categories. Across studies published or posted from 2024 to May 14, 2026, evaluation practices expanded rapidly while definitions, units of analysis, and reporting conventions remained fragmented. Evaluation coverage was concentrated in offline-only and simulated evaluation settings and was sparser in workflow-facing, prospective, and deployment-level settings, especially for retrieval-layer evaluation, fine-grained evidence verification, contradiction handling, formal safety-related evaluation, bias-control measures for LLM-as-judge evaluation, GraphRAG intermediate artifacts, and deployment-level monitoring. These findings support descriptive mapping of evaluation practices and suggest that clinical readiness claims are more interpretable when supported by layer-specific evidence [18,21,22].

### Comparison With Previous Work

Independent retrieval-layer evaluation was reported in only a minority of included studies, despite inference-time retrieval being a defining feature of all eligible systems. When retrieval is evaluated only through final outputs, output-level errors are more difficult to attribute to retrieval failures, evidence selection failures, or generation failures [12]. This limits failure-mode localization and weakens comparative interpretation across RAG pipelines. The practical consequence is that end-to-end improvements can be misattributed to retrieval changes when they may primarily reflect prompt design, decoding constraints, answer formatting, or evaluator sensitivity. Conversely, apparently weak performance can be driven by corpus segmentation or retrieval unit choices rather than by limitations in generation. Without retrieval-layer evaluation and clear specification of retrieval unit and granularity, claims about the causal role of retrieval in improving factual reliability remain difficult to substantiate. This interpretation is consistent with broader RAG evaluation literature, which emphasizes that hybrid systems require layer-aware assessment because retrieval relevance, generation quality, and answer faithfulness are related but nonidentical targets [18]. It also extends previous health care LLM reviews, which have documented fragmented practices but have focused less directly on component-level evaluation and failure-mode localization in retrieval-augmented systems [5,6].

The literature frequently reported evaluations labeled as grounding, faithfulness, citation and source correctness, or hallucination assessment, but these terms often referred to partially overlapping end points. Clear construct boundaries matter because these end points are not interchangeable and can yield conflicting conclusions when treated as substitutes [185]. Previous RAG and hallucination-evaluation literature has emphasized related constructs, but the health care studies mapped in this review often operationalized them with limited granularity or inconsistent terminology. Grounding concerns whether generated claims are supported by retrieved evidence available at inference time. Faithfulness concerns whether the response remains appropriately constrained by retrieved evidence, without introducing unsupported elaborations. Factuality concerns correctness with respect to an external reference standard that may include information not present in retrieved context. Citation and source correctness concerns whether cited sources actually support the local claims they are attached to, including placement, specificity, and claim-to-source alignment. Recent scholarship supports this distinction, showing that citation presence or citation plausibility is not equivalent to citation and source correctness, because apparently correct citations may still reflect post hoc rationalization rather than genuine evidence use [186]. Hallucination rubrics vary widely and may mix grounding violations, factuality errors, omissions, and miscalibrated certainty depending on rubric design [20,185]. Fine-grained evidence verification is therefore important because response-level assessment can mask localized unsupported claims, whereas claim-level, citation-level, or sentence-level verification can make evidence-linkage failures more visible [187].

Graph-structured systems were evaluated primarily through end-to-end output outcomes, while evaluation of intermediate artifacts, such as graph construction validity, retrieved paths or subgraphs, graph-derived summaries, and provenance-related interpretability, remained less consistently reported. This limits the ability to substantiate the primary motivations for graph structure, particularly claims about improved traceability and interpretability. The practical consequence is that graph-structured approaches can be judged using end points that do not test their stated advantages, which weakens interpretation of when graph structure provides measurable benefits and which graph components contribute to improvements or failures. Because a limited but expanding subset of studies met the minimum GraphRAG criterion, these findings should be interpreted as exploratory rather than definitive. When interpretability or provenance is presented as a rationale for graph-structured RAG, intermediate-artifact evaluation may help interpret the added value of GraphRAG beyond conventional RAG pipelines [15].

Evaluation-setting context is central to interpreting health care evaluation evidence. Most studies evaluated systems in offline-only settings, with vignette- or case-based testing used as an intermediate step in some work. Offline evaluation is essential for controlled iteration, yet it can underrepresent workflow constraints, incomplete context, time pressure, and local practice variation, all of which directly shape reliability and clinician reliance. This observation aligns with broader health care LLM reviews finding limited high-realism prospective evidence [5,6]. Previous health care AI evaluation guidance has similarly emphasized staged evaluation before clinical implementation, including escalation, oversight, governance, and monitoring requirements as systems move closer to clinical use [23]. In this review, the evidence-and-gap map showed sparse workflow-facing, prospective, and deployment-level coverage for fine-grained evidence verification, contradiction handling, safety-related assessment, human-evaluation reliability reporting, bias-control measures for LLM-as-judge evaluation, and GraphRAG intermediate-artifact evaluation. This pattern supports aligning evaluation expectations with the intended use context. Early-stage systems may reasonably begin with retrieval-layer testing, end-to-end task metrics, and evidence-linkage checks, whereas workflow-facing systems warrant added attention to safety monitoring, contradiction handling, escalation pathways, user interaction, and governance.

Human evaluation was reported in over half of studies, often involving clinicians or domain experts. However, IRR was reported in only a minority of human-evaluated studies. Limited reporting of rater expertise, training, and reliability restricts interpretation of end points such as usefulness, appropriateness, and safety, which are constructs sensitive to rubric framing and rater background. Without reliability evidence, apparent differences between systems may reflect measurement instability rather than robust behavioral differences [188]. LLM-as-judge evaluation was used in a subset of studies, yet bias-control measures for LLM-as-judge evaluation were reported in fewer than half of those studies [189]. This pattern suggests automated evaluation is being adopted for scalability with variable reporting of safeguards. Validity threats include prompt sensitivity [190], judge drift, correlated model errors, and reward hacking [191,192]. Safety-related evaluation also requires explicit operationalization because grounded or factually accurate responses can still be unsafe when they omit warnings, express inappropriate certainty, ignore patient-specific constraints, or fail under conflicting evidence [1,193-196].

### Implications for Evaluation and Reporting

Reporting of implementation-relevant study details varied across the included studies. In retrieval-augmented systems, incomplete reporting is particularly consequential because system behavior depends on corpus provenance and versioning, chunking policy, embedding and retrieval configurations, reranking settings, and citation policies [197]. A concrete consequence is that nominally similar systems may behave differently due to unreported corpus or indexing differences, while readers attribute differences to model choice or retrieval method. Improving reporting transparency therefore requires emphasizing RAG-specific determinants of behavior as first-class reporting items rather than as implementation details [8]. This observation strongly aligns with the movement toward LLM-specific reporting standards in biomedicine, such as TRIPOD-LLM [8], and emerging governance frameworks emphasizing continuous drift monitoring [198].

Based on the descriptive synthesis and the evidence-and-gap map, these findings point to several practice-oriented implications for future evaluation and reporting. Retrieval and generation may be more interpretable when evaluated as separable layers, particularly when retrieval is claimed to improve performance. Grounding, faithfulness, and citation and source correctness are more interpretable when explicitly defined and assessed at a stated verification unit. Contradiction handling, uncertainty communication, and formal safety-related evaluation are particularly relevant when systems are positioned for clinical or workflow-facing use. For human evaluation and LLM-as-judge evaluation, reporting safeguards that support measurement validity may improve interpretability, including rater expertise, IRR, evaluator identity, prompts or rubrics, calibration, and sensitivity analyses. Graph-structured systems should consider GraphRAG construction evaluation and intermediate-artifact evaluation when interpretability, provenance, or graph-based evidence organization is presented as a key contribution. These priorities are consistent with emerging reporting guidance for LLM studies, RAG failure-mode analyses, health care AI evaluation frameworks, and literature on LLM-as-judge evaluation and medical safety evaluation [1,11,22,23,189-197].

The synthesis-informed evaluation considerations proposed in this review should therefore be interpreted as a practice-oriented synthesis to support more transparent and setting-appropriate evaluation, rather than as a mandatory checklist or an ordinal maturity scale. These considerations are intended to help align evaluation intensity with intended use context, while allowing task- and context-specific adaptation [11,22,23,197,198].

### Limitations

This scoping review has limitations. First, the review focused on contemporary studies from 2024 onward, with searches conducted through May 14, 2026; earlier foundational work may therefore be underrepresented. The review was limited to English-language records, which may have excluded relevant studies reported in other languages. Second, consistent with scoping review methodology, the synthesis maps reported practices rather than estimating pooled effects, and formal risk-of-bias appraisal lay outside the review scope [24,25]. Third, findings depend on reporting completeness; procedures performed but left undescribed may be underestimated, and classification necessarily depended on study descriptions; information relevant to a field but not explicitly described was coded as not reported, and items structurally irrelevant to a study design or evaluation approach were coded as not applicable. Fourth, heterogeneity in tasks, corpora, including private and EHR-derived resources, and evaluation end points limited quantitative synthesis and supported the descriptive nature of the evidence-and-gap map. Finally, the identification of GraphRAG systems relied on prespecified operational criteria; studies may be classified differently under alternative definitions that use broader or narrower criteria for graph-structured inference-time retrieval and evidence assembly.

### Conclusions

This scoping review contributes to health care generative AI evaluation by mapping how RAG and GraphRAG systems are evaluated across retrieval, evidence linkage, safety, reporting, GraphRAG-specific artifacts, and evaluation-setting categories. Unlike previous reviews that broadly survey health care LLM applications or general NLP benchmarks, this study examines how evaluation is defined, operationalized, and reported within RAG and GraphRAG systems. The evidence-and-gap map shows areas of concentrated coverage and clinically relevant gaps, while the synthesis-informed evaluation considerations translate these gaps into practice-oriented evaluation considerations. These findings may inform more transparent, layer-specific, and safety-oriented evaluation planning for systems being considered for workflow-facing implementation [11,22,23,193,198].

## Supplementary material

10.2196/90046 Multimedia Appendix 1 Complete database-specific search strategies.

10.2196/90046 Multimedia Appendix 2 Study-level core coding tables and source data for the evidence-and-gap map.

10.2196/90046 Checklist 1 PRISMA-ScR checklist.

10.2196/90046 Checklist 2 PRISMA-S checklist.

## Abbreviations

AI: artificial intelligence
EHR: electronic health record
GraphRAG: graph-structured retrieval-augmented generation
IRR: interrater reliability
LLM: large language model
NLP: natural language processing
OSF: Open Science Framework
PCC: Population, Concept, and Context
PRISMA: Preferred Reporting Items for Systematic Reviews and Meta-Analyses
PRISMA-S: Preferred Reporting Items for Systematic Reviews and Meta-Analyses literature search extension
PRISMA-ScR: Preferred Reporting Items for Systematic Reviews and Meta-Analyses extension for Scoping Reviews
RAG: retrieval-augmented generation
