# Supplementary material for: Evaluation Methods for Inference-Time Retrieval-Augmented and Graph Retrieval-Augmented Large Language Models in Health Care: Scoping Review
Source: J Med Internet Res. 2026 Aug 3;28:e90046. doi: 10.2196/90046 (PMC13432247; doi:10.2196/90046)
Supplement: Multimedia Appendix 1 [file jmir-v28-e90046-s001.docx]

**Search Strategies**

# Full search strategies

**Note on preprint platforms.** Because advanced syntax and searchable fields differ across preprint platforms, the arXiv and medRxiv strategies were adapted to platform-supported search options. These searches were supplemented by backward and forward citation tracking to reduce the chance of missing eligible records.

**Overview. Searches were conducted on May 14, 2026 in bibliographic databases and preprint platforms selected to capture biomedical, interdisciplinary, engineering, and computing literature relevant to healthcare retrieval-augmented large language model (LLM) systems. Searches were limited to English-language records published from January 1, 2024 to May 14, 2026. Search strategies combined controlled vocabulary where supported and free-text terms for four concept blocks: (1) large language models, (2) inference-time retrieval augmentation, including graph-structured retrieval approaches, (3) healthcare context, and (4) evaluation-related terms. Database-specific syntax, searchable fields, and proximity operators were adapted to each platform.**

**Rationale for evaluation-related terms.** Because the review aimed to synthesize evaluation practices rather than all healthcare RAG applications, evaluation-related terms were included to improve precision for records explicitly reporting evaluation methods, benchmarking designs, or evidence-verification procedures. To mitigate the possibility that this design reduced sensitivity for studies whose evaluation components were not reflected in searchable fields, backward and forward citation tracking was additionally performed for all included studies.

Reporting note. Counts below are reported before deduplication. Search strings are reproduced as run on each platform, with minor formatting normalization for readability.

**PubMed (MEDLINE)**

| **Platform** | **PubMed (MEDLINE)** |
| --- | --- |
| **Date searched** | May 14, 2026 |
| **Publication date limits** | January 1, 2024 to May 14, 2026 |
| **Language limits** | English |
| **Records retrieved before deduplication** | 771 |

**Search strategy**

| ( "Large Language Models"[MeSH] OR "large language model*"[tiab] OR LLM[tiab] OR LLMs[tiab] OR "generative AI"[tiab] OR "generative artificial intelligence"[tiab] OR GPT[tiab] OR "GPT-4"[tiab] OR ChatGPT[tiab] OR Claude[tiab] OR Gemini[tiab] OR Llama[tiab] ) AND ( "retrieval-augmented generation"[tiab] OR "retrieval augmented generation"[tiab] OR RAG[tiab] OR ("retrieval"[tiab] AND augmented[tiab] AND LLM[tiab]) OR ("retrieval"[tiab] AND augmented[tiab] AND LLMs[tiab]) OR "retrieval based"[tiab] OR (knowledge[tiab] AND ground*[tiab]) OR (evidence[tiab] AND ground*[tiab]) OR (source[tiab] AND ground*[tiab]) OR GraphRAG[tiab] OR "graph rag"[tiab] OR ("graph"[tiab] AND based[tiab] AND RAG[tiab]) OR "graph-based retrieval"[tiab] OR "knowledge graph*"[tiab] OR ("knowledge graph*"[tiab] AND retriev*[tiab]) OR "graph retrieval"[tiab] OR (subgraph*[tiab] AND retriev*[tiab]) ) AND ( "Health"[MeSH] OR "Medicine"[MeSH] OR healthcare[tiab] OR "health care"[tiab] OR medical[tiab] OR clinical[tiab] OR biomedical[tiab] OR hospital*[tiab] OR patient*[tiab] OR clinician*[tiab] OR physician*[tiab] OR nurs*[tiab] OR "electronic health record*"[tiab] OR EHR[tiab] OR "electronic medical record*"[tiab] OR EMR[tiab] OR "clinical decision support"[tiab] OR CDSS[tiab] ) AND ( evaluat*[tiab] OR assess*[tiab] OR validat*[tiab] OR benchmark*[tiab] OR perform*[tiab] OR compar*[tiab] OR metric*[tiab] OR "human evaluation"[tiab] OR "expert evaluation"[tiab] OR "clinician evaluation"[tiab] OR "error analysis"[tiab] OR faithful*[tiab] OR ground*[tiab] OR hallucinat*[tiab] OR (citation*[tiab] AND correct*[tiab]) OR "claim verification"[tiab] OR "fact checking"[tiab] ) NOT ( review[pt] OR "systematic review"[tiab] OR "scoping review"[tiab] OR "meta-analysis"[tiab] OR "narrative review"[tiab] ) |
| --- |

**Web of Science Core Collection**

| **Platform** | **Web of Science Core Collection** |
| --- | --- |
| **Date searched** | May 14, 2026 |
| **Publication date limits** | January 1, 2024 to May 14, 2026 |
| **Language limits** | English |
| **Records retrieved before deduplication** | 579 |

**Search strategy**

| TS=( ( "large language model*" OR LLM OR LLMs OR "generative AI" OR "generative artificial intelligence" OR GPT OR "GPT-4" OR ChatGPT OR Claude OR Gemini OR Llama ) AND ( "retrieval-augmented generation" OR "retrieval augmented generation" OR RAG OR ("retrieval" NEAR/2 augmented) OR "retrieval based" OR (knowledge NEAR/2 ground*) OR (evidence NEAR/2 ground*) OR (source NEAR/2 ground*) OR GraphRAG OR "graph rag" OR ("graph-based" NEAR/2 RAG) OR "graph-based retrieval" OR "knowledge graph*" OR ("knowledge graph*" NEAR/3 retriev*) OR "graph retrieval" OR (subgraph* NEAR/3 retriev*) ) AND ( healthcare OR "health care" OR medical OR clinical OR biomedical OR hospital* OR patient* OR clinician* OR physician* OR nurs* OR "electronic health record*" OR EHR OR "electronic medical record*" OR EMR OR "clinical decision support" OR CDSS ) AND ( evaluat* OR assess* OR validat* OR benchmark* OR perform* OR compar* OR metric* OR "human evaluation" OR "expert evaluation" OR "clinician evaluation" OR "error analysis" OR faithful* OR ground* OR hallucinat* OR (citation* NEAR/2 correct*) OR "claim verification" OR "fact checking" ) ) NOT TS=( "systematic review" OR "scoping review" OR "meta-analysis" OR "narrative review" ) |
| --- |

**IEEE Xplore**

| **Platform** | **IEEE Xplore** |
| --- | --- |
| **Date searched** | May 14, 2026 |
| **Publication date limits** | January 1, 2024 to May 14, 2026 |
| **Language limits** | English |
| **Records retrieved before deduplication** | 409 |

**Search strategy**

| ( ("Document Title":"large language model" OR "Document Title":"large language models" OR "Abstract":"large language model" OR "Abstract":"large language models" OR "Author Keywords":"large language model" OR "Author Keywords":"large language models" OR "Abstract":LLM OR "Abstract":LLMs OR "Abstract":"generative AI" OR "Abstract":"generative artificial intelligence" OR "Abstract":GPT OR "Abstract":"GPT-4" OR "Abstract":ChatGPT OR "Abstract":Claude OR "Abstract":Gemini OR "Abstract":Llama) AND ("Abstract":"retrieval-augmented generation" OR "Abstract":"retrieval augmented generation" OR "Abstract":RAG OR "Abstract":"retrieval based" OR "Abstract":"knowledge grounded" OR "Abstract":"evidence grounded" OR "Abstract":"source grounded" OR "Abstract":GraphRAG OR "Abstract":"graph rag" OR "Abstract":"graph-based retrieval" OR "Abstract":"knowledge graph" OR "Abstract":"knowledge graphs" OR "Abstract":"graph retrieval" OR "Abstract":"subgraph retrieval") AND ("Abstract":healthcare OR "Abstract":"health care" OR "Abstract":medical OR "Abstract":clinical OR "Abstract":biomedical OR "Abstract":hospital OR "Abstract":patient OR "Abstract":patients OR "Abstract":clinician OR "Abstract":clinicians OR "Abstract":physician OR "Abstract":physicians OR "Abstract":nurse OR "Abstract":nurses OR "Abstract":"electronic health record" OR "Abstract":"electronic health records" OR "Abstract":EHR OR "Abstract":"electronic medical record" OR "Abstract":"electronic medical records" OR "Abstract":EMR OR "Abstract":"clinical decision support" OR "Abstract":CDSS) AND ("Abstract":evaluat* OR "Abstract":assess* OR "Abstract":validat* OR "Abstract":benchmark* OR "Abstract":perform* OR "Abstract":compar* OR "Abstract":metric* OR "Abstract":"human evaluation" OR "Abstract":"expert evaluation" OR "Abstract":"clinician evaluation" OR "Abstract":"error analysis" OR "Abstract":faithful* OR "Abstract":ground* OR "Abstract":hallucinat* OR "Abstract":"citation correctness" OR "Abstract":"claim verification" OR "Abstract":"fact checking") ) NOT ( "Abstract":"systematic review" OR "Abstract":"scoping review" OR "Abstract":"meta-analysis" OR "Abstract":"narrative review" ) |
| --- |

**ACM Digital Library**

| **Platform** | **ACM Digital Library** |
| --- | --- |
| **Date searched** | May 14, 2026 |
| **Publication date limits** | January 1, 2024 to May 14, 2026 |
| **Language limits** | English |
| **Records retrieved before deduplication** | 2271 |

**Search strategy**

| ( ("large language model" OR "large language models" OR LLM OR LLMs OR "generative AI" OR "generative artificial intelligence" OR GPT OR "GPT-4" OR ChatGPT OR Claude OR Gemini OR Llama) AND ("retrieval-augmented generation" OR "retrieval augmented generation" OR RAG OR "retrieval based" OR "knowledge grounded" OR "evidence grounded" OR "source grounded" OR GraphRAG OR "graph rag" OR "graph-based retrieval" OR "knowledge graph" OR "knowledge graphs" OR "graph retrieval" OR "subgraph retrieval") AND (healthcare OR "health care" OR medical OR clinical OR biomedical OR hospital* OR patient* OR clinician* OR physician* OR nurs* OR "electronic health record*" OR EHR OR "electronic medical record*" OR EMR OR "clinical decision support" OR CDSS) AND (evaluat* OR assess* OR validat* OR benchmark* OR perform* OR compar* OR metric* OR "human evaluation" OR "expert evaluation" OR "clinician evaluation" OR "error analysis" OR faithful* OR ground* OR hallucinat* OR "citation correctness" OR "claim verification" OR "fact checking") ) AND NOT ("systematic review" OR "scoping review" OR "meta-analysis" OR "narrative review") |
| --- |

**arXiv**

| **Platform** | **arXiv** |
| --- | --- |
| **Date searched** | May 14, 2026 |
| **Publication date limits** | January 1, 2024 to May 14, 2026 |
| **Language limits** | English |
| **Records retrieved before deduplication** | 404 |

**Search strategy**

| Abstract: ("large language model*" OR LLM OR "generative AI" OR "generative artificial intelligence" OR "GPT-4" OR ChatGPT OR Claude OR Gemini OR Llama) AND Abstract: ("retrieval-augmented generation" OR "retrieval augmented generation" OR RAG OR GraphRAG OR "graph-based retrieval") AND Abstract: (healthcare OR "health care" OR medical OR clinical OR biomedical OR hospital* OR patient* OR clinician* OR physician* OR nurs* OR "electronic health record*" OR EHR OR "electronic medical record*" OR EMR OR "clinical decision support" OR CDSS) AND Abstract: (evaluat* OR assess* OR validat* OR benchmark* OR metric* OR faithful* OR hallucinat* OR "human evaluation" OR "expert evaluation" OR "clinician evaluation" OR "error analysis" OR "claim verification" OR "fact checking") NOT Title: ("systematic review" OR "scoping review" OR "meta-analysis" OR "narrative review") |
| --- |

**medRxiv**

| **Platform** | **medRxiv** |
| --- | --- |
| **Date searched** | May 14, 2026 |
| **Publication date limits** | January 1, 2024 to May 14, 2026 |
| **Language limits** | English |
| **Records retrieved before deduplication** | 190 |

**Search strategy**

| ("large language model" OR LLM OR ChatGPT) AND ("retrieval-augmented generation" OR "retrieval augmented generation")  AND Abstract or Title: healthcare medical clinical biomedical hospital patient clinician physician nurse EHR EMR CDSS  AND Full Text or Abstract or Title: evaluation evaluate validation validate benchmark metric faithful faithfulness hallucination hallucinations |
| --- |

**Abbreviations**

**CDSS**, clinical decision support system; **EHR**, electronic health record; **EMR**, electronic medical record; **LLM**, large language model; **MeSH**, Medical Subject Headings; **PRISMA-S**, Preferred Reporting Items for Systematic Reviews and Meta-Analyses literature search extension; **RAG**, retrieval-augmented generation.
