# Supplementary material for: Evaluation Methods for Inference-Time Retrieval-Augmented and Graph Retrieval-Augmented Large Language Models in Health Care: Scoping Review
Source: J Med Internet Res. 2026 Aug 3;28:e90046. doi: 10.2196/90046 (PMC13432247; doi:10.2196/90046)
Supplement: Multimedia Appendix 2 [file jmir-v28-e90046-s002.docx]

**Multimedia Appendix 2. Study-Level Core Coding Tables and Figure Source Data**

**Coding conventions**

This appendix provides the study-level core coding tables and source data for Figure 3 for the 157 included studies. Multi-label fields are separated by semicolons. "Not reported" indicates that an item was applicable but was not explicitly described in the source study. "Not applicable" indicates that an item was structurally irrelevant to the study design or evaluation approach. Application task categories, evaluation-setting categories, and evaluation domains were coded as nonmutually exclusive. For each distinct grounding or evidence-linkage evaluation component, the most granular verification unit explicitly described was recorded; therefore, study-level summary categories for verification granularity were also nonmutually exclusive. LLM-as-judge bias-control measures were considered applicable only when an LLM was used as an evaluator.

Abbreviations: EHR, electronic health record; GraphRAG, graph-structured retrieval-augmented generation; IRB, institutional review board; LLM, large language model; RAG, retrieval-augmented generation.

**Table S1. Study characteristics and application/evaluation-setting coding**

| **Study No.** | **Citation** | **Application task categories** | **Evaluation-setting categories** | **Knowledge source category** | **Retrieval corpus explicitly EHR-related** |
| --- | --- | --- | --- | --- | --- |
| 1 | Karim, A H M Rezaul (2025) | Clinical question answering; Medical visual question answering | Offline-only evaluation | Public or public-mixed knowledge sources | No |
| 2 | Wada, Akihiko (2025) | Clinical decision support | Offline-only evaluation; Simulated vignette or case evaluation | Public or public-mixed knowledge sources | No |
| 3 | Fink, Anna (2025) | Clinical decision support | Offline-only evaluation; Simulated vignette or case evaluation | Public or public-mixed knowledge sources | No |
| 4 | Kelly, Anthony (2025) | Clinical question answering; Patient or caregiver education | Offline-only evaluation; Simulated vignette or case evaluation | Public or public-mixed knowledge sources | No |
| 5 | Su, Audrey Y. (2025) | Clinical decision support | Offline-only evaluation | Public or public-mixed knowledge sources | No |
| 6 | Chung Man Ho (2025) | Clinical question answering; Patient or caregiver education | Workflow pilot or user study | Private institutional knowledge sources, including mixed sources | No |
| 7 | Gan, Chunjing (2025) | Clinical question answering | Offline-only evaluation | Public or public-mixed knowledge sources | No |
| 8 | Tarabanis, Constantine (2026) | Clinical question answering | Offline-only evaluation | Public or public-mixed knowledge sources | No |
| 9 | de Jesus, Davi dos Reis (2025) | Clinical decision support; Patient or caregiver education | Offline-only evaluation; Simulated vignette or case evaluation | Public or public-mixed knowledge sources | No |
| 10 | David Baur (2025) | Clinical question answering; Patient or caregiver education | Workflow pilot or user study; Real-world deployment or postdeployment monitoring | Public or public-mixed knowledge sources | No |
| 11 | Steybe, David (2025) | Clinical question answering | Offline-only evaluation | Public or public-mixed knowledge sources | No |
| 12 | Yu, Deshui (2025) | Clinical question answering | Offline-only evaluation | Public or public-mixed knowledge sources | No |
| 13 | Dingqiao Wang (2024) | Clinical question answering; Patient or caregiver education | Offline-only evaluation | Public or public-mixed knowledge sources | No |
| 14 | Dongchen Li (2026) | Clinical decision support; Summary or report generation | Offline-only evaluation | Private institutional knowledge sources, including mixed sources | Yes |
| 15 | Busch (2025) | Clinical question answering; Patient or caregiver education | Prospective clinical study; Workflow pilot or user study | Public or public-mixed knowledge sources | No |
| 16 | Hyunjae Kim (2025) | Clinical question answering; Clinical decision support | Offline-only evaluation | Public or public-mixed knowledge sources | No |
| 17 | Jiwoong Sohn (2025) | Clinical question answering | Offline-only evaluation | Public or public-mixed knowledge sources | No |
| 18 | Junde Wu (2025) | Clinical question answering | Offline-only evaluation | Private institutional knowledge sources, including mixed sources | No |
| 19 | Justice Ou (2025) | Clinical question answering; Clinical decision support; Summary or report generation | Offline-only evaluation | Private institutional knowledge sources, including mixed sources | Yes |
| 20 | Karthik Soman (2024) | Clinical question answering | Offline-only evaluation | Public or public-mixed knowledge sources | No |
| 21 | Krzysztof Wołk (2025) | Clinical decision support | Offline-only evaluation; Simulated vignette or case evaluation | Public or public-mixed knowledge sources | No |
| 22 | Jonggwon Park (2026) | Clinical question answering | Offline-only evaluation; Simulated vignette or case evaluation | Public or public-mixed knowledge sources | No |
| 23 | Leon Garza (2025) | Clinical decision support | Offline-only evaluation | Private institutional knowledge sources, including mixed sources | Yes |
| 24 | Linus Stuhlmann (2025) | Clinical question answering | Offline-only evaluation | Public or public-mixed knowledge sources | No |
| 25 | Liping Xiong (2025) | Clinical question answering | Offline-only evaluation | Private institutional knowledge sources, including mixed sources | Yes |
| 26 | Liwen Sun (2025) | Summary or report generation | Offline-only evaluation | Public or public-mixed knowledge sources | No |
| 27 | M. Berkan Sesen (2025) | Clinical decision support | Offline-only evaluation; Simulated vignette or case evaluation | Public or public-mixed knowledge sources | No |
| 28 | Madeline K. Moureau (2025) | Clinical question answering; Patient or caregiver education | Offline-only evaluation | Public or public-mixed knowledge sources | No |
| 29 | Matthew Lewis (2025) | Clinical question answering; Clinical decision support | Offline-only evaluation | Public or public-mixed knowledge sources | No |
| 30 | Michael Welsh (2025) | Clinical question answering | Offline-only evaluation | Public or public-mixed knowledge sources | No |
| 31 | Mohammad Alkhalaf (2024) | Summary or report generation | Offline-only evaluation | Private institutional knowledge sources, including mixed sources | Yes |
| 32 | Mohammad Reza Rezaei (2025) | Clinical question answering | Offline-only evaluation | Public or public-mixed knowledge sources | No |
| 33 | Namrye Son (2025) | Administrative/operational question answering; EMR manual support | Offline-only evaluation | Private institutional knowledge sources, including mixed sources | No |
| 34 | Nuran Abdullayev (2025) | Clinical decision support | Offline-only evaluation; Simulated vignette or case evaluation | Public or public-mixed knowledge sources | No |
| 35 | Valan, P. V. P. (2025) | Patient or caregiver education | Offline-only evaluation | Public or public-mixed knowledge sources | No |
| 36 | Peng Xia (2024) | Summary or report generation | Offline-only evaluation | Public or public-mixed knowledge sources | No |
| 37 | Philip DiGiacomo (2025) | Clinical question answering | Offline-only evaluation | Public or public-mixed knowledge sources | No |
| 38 | Rong Chen (2025) | Clinical decision support | Offline-only evaluation | Public or public-mixed knowledge sources | No |
| 39 | Sebastian Wind (2025) | Clinical question answering | Offline-only evaluation; Simulated vignette or case evaluation | Public or public-mixed knowledge sources | No |
| 40 | Sheng-Ming Kuo (2025) | Summary or report generation | Workflow pilot or user study | Private institutional knowledge sources, including mixed sources | Yes |
| 41 | Sichu Liang (2025) | Clinical question answering; Clinical decision support | Offline-only evaluation | Public or public-mixed knowledge sources | Yes |
| 42 | Siyang Liu (2025) | Patient or caregiver education; Clinical question answering | Workflow pilot or user study | Public or public-mixed knowledge sources | No |
| 43 | Skatje Myers (2025) | Clinical decision support | Offline-only evaluation | Private institutional knowledge sources, including mixed sources | Yes |
| 44 | Tasnimul Hassan (2025) | Clinical question answering | Offline-only evaluation | Public or public-mixed knowledge sources | No |
| 45 | Tharun Sekar (2025) | Clinical question answering | Offline-only evaluation | Public or public-mixed knowledge sources | No |
| 46 | Vijaya Parameswaran (2025) | Clinical question answering; Patient or caregiver education | Offline-only evaluation | Public or public-mixed knowledge sources | No |
| 47 | Wenchuan Zhang (2026) | Clinical question answering; Medical visual question answering | Offline-only evaluation | Public or public-mixed knowledge sources | No |
| 48 | Xiaowei Xu (2025) | Clinical question answering; Specialty education/training | Offline-only evaluation | Public or public-mixed knowledge sources | No |
| 49 | Xuanzhao Dong (2025) | Clinical question answering | Offline-only evaluation | Public or public-mixed knowledge sources | No |
| 50 | Xuejiao Zhao (2025) | Clinical question answering; Clinical decision support | Offline-only evaluation | Private institutional knowledge sources, including mixed sources | Yes |
| 51 | Xueren Ge (2025) | Clinical question answering | Offline-only evaluation | Private institutional knowledge sources, including mixed sources | No |
| 52 | Yash Mali (2025) | Clinical decision support | Offline-only evaluation; Simulated vignette or case evaluation | Public or public-mixed knowledge sources | No |
| 53 | Yi Yu (2025) | Clinical decision support | Offline-only evaluation | Public or public-mixed knowledge sources | No |
| 54 | Yu He Ke (2025) | Clinical question answering | Offline-only evaluation | Private institutional knowledge sources, including mixed sources | No |
| 55 | Yuelyu Ji (2025) | Clinical question answering | Offline-only evaluation | Public or public-mixed knowledge sources | No |
| 56 | Zhongzhen Huang (2024) | Clinical question answering; Clinical decision support | Offline-only evaluation | Public or public-mixed knowledge sources | No |
| 57 | Zifeng Wang (2025) | Summary or report generation | Offline-only evaluation | Public or public-mixed knowledge sources | No |
| 58 | Ziyu Wang (2025) | Clinical question answering | Offline-only evaluation | Public or public-mixed knowledge sources | No |
| 59 | Xu, Ruiyu (2024) | Clinical question answering; Patient or caregiver education | Offline-only evaluation | Public or public-mixed knowledge sources | No |
| 60 | Luo, Ming-Jie (2024) | Clinical question answering; Clinical decision support | Offline-only evaluation | Private institutional knowledge sources, including mixed sources | No |
| 61 | Zhang, Gongbo (2025) | Clinical question answering | Offline-only evaluation | Public or public-mixed knowledge sources | No |
| 62 | Low, Yen Sia (2025) | Clinical question answering; Clinical decision support | Offline-only evaluation | Public or public-mixed knowledge sources | No |
| 63 | Ge, Jin (2025) | Clinical question answering; Clinical decision support | Offline-only evaluation | Public or public-mixed knowledge sources | No |
| 64 | Zakka, Cyril (2024) | Clinical question answering; Clinical decision support | Offline-only evaluation | Public or public-mixed knowledge sources | No |
| 65 | Tozuka, Ryota (2025) | Clinical decision support | Offline-only evaluation; Simulated vignette or case evaluation | Public or public-mixed knowledge sources | No |
| 66 | Hewitt, Katherine J (2024) | Diagnosis support | Offline-only evaluation; Simulated vignette or case evaluation | Public or public-mixed knowledge sources | No |
| 67 | Wang, Dingqiao (2025) | Clinical question answering | Offline-only evaluation | Public or public-mixed knowledge sources | No |
| 68 | Xu, Yupeng (2025) | Summary or report generation | Offline-only evaluation | Private institutional knowledge sources, including mixed sources | Yes |
| 69 | Tung, Joshua Yi Min (2025) | Clinical decision support | Offline-only evaluation; Simulated vignette or case evaluation | Public or public-mixed knowledge sources | No |
| 70 | Zhang, Chi (2025) | Clinical question answering; Clinical decision support | Workflow pilot or user study | Public or public-mixed knowledge sources | No |
| 71 | Fanelli, Francesco (2025) | Clinical question answering | Offline-only evaluation | Public or public-mixed knowledge sources | No |
| 72 | Masanneck, Lars (2025a) | Clinical question answering; Clinical decision support | Offline-only evaluation; Simulated vignette or case evaluation | Public or public-mixed knowledge sources | No |
| 73 | Qimin Yang (2025) | Clinical question answering; Clinical decision support | Offline-only evaluation; Simulated vignette or case evaluation | Private institutional knowledge sources, including mixed sources | No |
| 74 | Marius Vach (2025) | Clinical question answering; Clinical decision support | Offline-only evaluation | Public or public-mixed knowledge sources | No |
| 75 | Masanneck, Lars (2025b) | Clinical question answering; Clinical decision support | Offline-only evaluation; Simulated vignette or case evaluation | Public or public-mixed knowledge sources | No |
| 76 | Yusuke Fukui (2025) | Clinical question answering | Offline-only evaluation | Private institutional knowledge sources, including mixed sources | No |
| 77 | Hangyu Sha (2025) | Patient or caregiver education | Offline-only evaluation | Public or public-mixed knowledge sources | No |
| 78 | Owoyemi, Joshua (2025) | Clinical question answering; Clinical decision support | Workflow pilot or user study | Public or public-mixed knowledge sources | No |
| 79 | Tata, Venkatesh (2025) | Clinical question answering; Clinical decision support | Offline-only evaluation; Simulated vignette or case evaluation | Private institutional knowledge sources, including mixed sources | No |
| 80 | Tayebi Arasteh, Soroosh (2025) | Clinical question answering; Clinical decision support | Offline-only evaluation; Simulated vignette or case evaluation | Public or public-mixed knowledge sources | No |
| 81 | Das, Sudeshna (2025) | Clinical question answering | Offline-only evaluation | Public or public-mixed knowledge sources | No |
| 82 | Hai Li (2025) | Public health fact-checking | Offline-only evaluation | Public or public-mixed knowledge sources | No |
| 83 | Shin, Minjeong (2025) | Clinical decision support; Patient or caregiver education | Offline-only evaluation; Simulated vignette or case evaluation | Public or public-mixed knowledge sources | No |
| 84 | Aguzzi, Gianluca (2025) | Patient or caregiver education | Offline-only evaluation | Public or public-mixed knowledge sources | No |
| 85 | Zhou, Qingqing (2024) | Clinical question answering; Patient or caregiver education | Offline-only evaluation | Public or public-mixed knowledge sources | No |
| 86 | Aminan, Mohammad (2025) | Clinical question answering; Clinical decision support | Offline-only evaluation; Simulated vignette or case evaluation | Public or public-mixed knowledge sources | No |
| 87 | Hsu, Hsin-Ling (2025) | Clinical decision support; Summary or report generation | Offline-only evaluation | Private institutional knowledge sources, including mixed sources | Yes |
| 88 | Kresevic, Simone (2024) | Clinical question answering; Clinical decision support | Offline-only evaluation; Simulated vignette or case evaluation | Public or public-mixed knowledge sources | No |
| 89 | Kang, Bongsu (2024) | Clinical question answering | Offline-only evaluation | Public or public-mixed knowledge sources | No |
| 90 | AlSammarraie, AlHasan (2025) | Patient or caregiver education | Offline-only evaluation | Public or public-mixed knowledge sources | No |
| 91 | Nandy, Gargi (2025) | Patient or caregiver education | Workflow pilot or user study | Public or public-mixed knowledge sources | No |
| 92 | Hetz, M. J. (2024) | Clinical question answering; Clinical decision support | Offline-only evaluation | Public or public-mixed knowledge sources | No |
| 93 | Ong, Chin Siang (2024) | Clinical decision support; Summary or report generation | Offline-only evaluation; Simulated vignette or case evaluation | Public or public-mixed knowledge sources | No |
| 94 | Nicolas Carl (2026) | Clinical question answering; Clinical decision support | Simulated vignette or case evaluation; Workflow pilot or user study | Public or public-mixed knowledge sources | No |
| 95 | Tytler, Kolawole (2025) | Clinical question answering; Clinical decision support | Workflow pilot or user study; Real-world deployment or postdeployment monitoring | Public or public-mixed knowledge sources | No |
| 96 | Wu, Yue (2025) | Patient or caregiver education; Clinical question answering | Workflow pilot or user study; Prospective clinical study | Public or public-mixed knowledge sources | No |
| 97 | Kim, Seonok (2025) | Clinical question answering | Offline-only evaluation | Public or public-mixed knowledge sources | No |
| 98 | Hasan, Md. Mehedi (2025) | Clinical decision support | Offline-only evaluation; Simulated vignette or case evaluation | Public or public-mixed knowledge sources | No |
| 99 | Long, Yongchao (2025) | Clinical question answering; Clinical decision support | Offline-only evaluation; Simulated vignette or case evaluation | Private institutional knowledge sources, including mixed sources | No |
| 100 | Zhang, Jinning (2026) | Clinical question answering | Offline-only evaluation; Simulated vignette or case evaluation | Public or public-mixed knowledge sources | No |
| 101 | Ryan, Jessica (2026) | Clinical question answering | Offline-only evaluation | Public or public-mixed knowledge sources | No |
| 102 | Yang, Weiqin (2026) | Medical visual question answering; Summary or report generation; Medical imaging | Offline-only evaluation | Public or public-mixed knowledge sources | No |
| 103 | Lorenzo, Luis (2026) | Clinical decision support; Summary or report generation | Offline-only evaluation | Private institutional knowledge sources, including mixed sources | No |
| 104 | Li, Zheng (2026) | Clinical decision support | Offline-only evaluation | Public or public-mixed knowledge sources | No |
| 105 | Samanta, Himadri (2026) | Summary or report generation | Offline-only evaluation | Public or public-mixed knowledge sources | No |
| 106 | Chan, Richard Wai Cheung (2026) | Clinical question answering; Patient or caregiver education | Offline-only evaluation; Simulated vignette or case evaluation | Public or public-mixed knowledge sources | No |
| 107 | Chen, Xupeng (2026) | Clinical question answering; Medical visual question answering | Offline-only evaluation | Public or public-mixed knowledge sources | No |
| 108 | Khosa, Thokozile (2026) | Clinical question answering | Offline-only evaluation | Public or public-mixed knowledge sources | No |
| 109 | Abdul Rehman Akbar (2026) | Clinical question answering; Clinical decision support; Summary or report generation | Offline-only evaluation | Private institutional knowledge sources, including mixed sources | No |
| 110 | Jianmin Li (2026) | Clinical question answering; Clinical decision support | Offline-only evaluation; Simulated vignette or case evaluation | Public or public-mixed knowledge sources | No |
| 111 | Haoqing Li (2025) | Clinical question answering; Clinical decision support; Medical visual question answering | Offline-only evaluation; Simulated vignette or case evaluation | Private institutional knowledge sources, including mixed sources | No |
| 112 | Zhe Chen (2025b) | Medical visual question answering; Summary or report generation; Medical imaging | Offline-only evaluation | Public or public-mixed knowledge sources | No |
| 113 | Wenhao Li (2025) | Clinical decision support | Offline-only evaluation | Public or public-mixed knowledge sources | No |
| 114 | Mohammad Shoaib Ansari (2025) | Clinical decision support; Summary or report generation | Offline-only evaluation | Private institutional knowledge sources, including mixed sources | Yes |
| 115 | Peng Xia (2025) | Medical visual question answering; Summary or report generation; Medical imaging | Offline-only evaluation | Public or public-mixed knowledge sources | No |
| 116 | Yingpeng Ning (2025) | Clinical question answering | Offline-only evaluation | Public or public-mixed knowledge sources | No |
| 117 | Houman Kazemzadeh (2025) | Clinical question answering | Offline-only evaluation | Public or public-mixed knowledge sources | No |
| 118 | Yingjian Chen (2025) | Clinical question answering | Offline-only evaluation | Public or public-mixed knowledge sources | No |
| 119 | Han, Zihan (2025) | Clinical decision support; Summary or report generation | Offline-only evaluation | Private institutional knowledge sources, including mixed sources | Yes |
| 120 | Garapati, Keerthana (2025) | Summary or report generation | Offline-only evaluation | Clinical data (de-identified EHR notes from MIMIC-III) | Yes |
| 121 | Guo, Zhijun (2025) | Patient or caregiver education; Mental health screening | Workflow pilot or user study | Public or public-mixed knowledge sources | No |
| 122 | Zhu, Yu (2026) | Clinical decision support; Summary or report generation | Offline-only evaluation | Private institutional knowledge sources, including mixed sources | No |
| 123 | Mo, Gao (2026) | Administrative or operational support; Patient or caregiver education | Workflow pilot or user study | Private institutional knowledge sources, including mixed sources | No |
| 124 | Shahnawaz, Amna (2026) | Patient or caregiver education | Workflow pilot or user study; Real-world deployment or postdeployment monitoring | Public or public-mixed knowledge sources | No |
| 125 | Ahalpara, Trishala Jayesh (2025) | Patient or caregiver education | Workflow pilot or user study | Public or public-mixed knowledge sources | No |
| 126 | Boumans, Roel (2025) | Patient or caregiver education | Workflow pilot or user study | Public or public-mixed knowledge sources | No |
| 127 | Parmanto, Bambang (2024) | Patient or caregiver education | Offline-only evaluation | Public or public-mixed knowledge sources | No |
| 128 | Hang, Ching Nam (2025) | Public health fact-checking | Offline-only evaluation | Graph-structured knowledge sources | No |
| 129 | Gu, Difei (2025) | Summary or report generation; Medical imaging | Offline-only evaluation | Clinical data | No |
| 130 | Yi, Ziruo (2025) | Summary or report generation | Offline-only evaluation | Public or public-mixed knowledge sources | No |
| 131 | Bang, Byeonghun (2026) | Clinical question answering; Clinical decision support | Offline-only evaluation | Public or public-mixed knowledge sources | No |
| 132 | Ting, Lo Pang-Yun (2025) | Summary or report generation | Offline-only evaluation | Public or public-mixed knowledge sources | Yes |
| 133 | Johno, Hisashi (2026) | Clinical decision support | Offline-only evaluation; Simulated vignette or case evaluation | Public or public-mixed knowledge sources | No |
| 134 | He, Jinglin (2025) | Clinical question answering; Clinical decision support | Offline-only evaluation | Graph-structured knowledge sources | No |
| 135 | Zhao, Yi-Fei (2025) | Patient or caregiver education | Offline-only evaluation; Simulated vignette or case evaluation | Public or public-mixed knowledge sources | No |
| 136 | Madrid-García, Alfredo (2025) | Clinical question answering; Clinical decision support | Offline-only evaluation | Public or public-mixed knowledge sources | No |
| 137 | Saidu, Fatima (2026) | Clinical decision support | Simulated vignette or case evaluation; Workflow pilot or user study | Graph-structured knowledge sources | No |
| 138 | Felde, Sabine (2026) | Clinical decision support; Clinical question answering | Offline-only evaluation; Simulated vignette or case evaluation | Public or public-mixed knowledge sources | No |
| 139 | Jeon, Yaejin (2026) | Clinical question answering; Clinical decision support | Offline-only evaluation | Public or public-mixed knowledge sources | No |
| 140 | Kang et al. (2026) | Clinical decision support; Clinical reasoning | Offline-only evaluation | Public or public-mixed knowledge sources | No |
| 141 | Komenda et al. (2026) | Clinical question answering; Clinical decision support | Offline-only evaluation | Public or public-mixed knowledge sources | No |
| 142 | Wang et al. (2026) | Patient or caregiver education; Public health education | Offline-only evaluation | Public or public-mixed knowledge sources | No |
| 143 | Zhang et al. (2026) | Clinical question answering; Clinical decision support | Offline-only evaluation | Public or public-mixed knowledge sources | No |
| 144 | Saadi, Soroush Baseri (2026) | Clinical decision support; Diagnosis support | Offline-only evaluation; Simulated vignette or case evaluation | Public or public-mixed knowledge sources | No |
| 145 | Song, Jong Keon (2026) | Clinical question answering; Clinical decision support | Offline-only evaluation | Graph-structured knowledge sources; Public or public-mixed knowledge sources | No |
| 146 | Kabak, Yildiray (2025) | Clinical decision support | Offline-only evaluation | Clinical data plus public or public-mixed knowledge sources | Yes |
| 147 | Ma, Jiaqing (2026) | Summary or report generation; Medical imaging | Offline-only evaluation | Graph-structured knowledge sources; Public or public-mixed knowledge sources | No |
| 148 | Anonymous ACL submission (2026) | Medical report generation; Medical imaging | Offline-only evaluation | Public or public-mixed knowledge sources; Multimodal evidence | No |
| 149 | Garcia-Font, Marc (2026) | Clinical question answering; Diagnosis support | Offline-only evaluation | Public or public-mixed knowledge sources | No |
| 150 | Wong, Hang Sheung (2026) | Clinical decision support; Triage | Offline-only evaluation | Clinical data plus public or public-mixed knowledge sources | Yes |
| 151 | Thio et al. (2026) | Clinical question answering; Clinical information retrieval | Offline-only evaluation | Graph-structured knowledge sources; Clinical data | Yes |
| 152 | Zaki et al. (2026) | Clinical decision support | Offline-only evaluation; Simulated vignette or case evaluation | Public or public-mixed knowledge sources | Yes |
| 153 | Liu, Haixiao (2026) | Clinical decision support | Offline-only evaluation | Clinical data plus public or public-mixed knowledge sources | Yes |
| 154 | Kim, DaeHo (2026) | Clinical question answering; Biomedical question answering | Offline-only evaluation | Graph-structured knowledge sources; Public or public-mixed knowledge sources | No |
| 155 | Lopez, Ivan (2025) | Biomedical information extraction; Clinical information extraction | Offline-only evaluation | Clinical data | Yes |
| 156 | Nanua, Suparna (2025) | Clinical question answering; Clinical decision support; Administrative or operational support | Offline-only evaluation; Simulated vignette or case evaluation | Public or public-mixed knowledge sources | No |
| 157 | Xie, Wenbo (2026) | Clinical decision support; Mental health triage | Offline-only evaluation; Simulated vignette or case evaluation | Clinical data plus public or public-mixed knowledge sources | No |

**Table S2. Retrieval-layer, grounding, and evidence-linkage evaluation coding**

| **Study No.** | **Citation** | **Retrieval-layer evaluation** | **Retrieval metric family or families** | **Grounding and faithfulness evaluation** | **Evidence-verification unit** | **Fine-grained evidence verification** | **Citation and source correctness evaluation** | **Conflict or contradiction handling evaluation** |
| --- | --- | --- | --- | --- | --- | --- | --- | --- |
| 1 | Karim, A H M Rezaul (2025) | No | Not applicable | No | Not applicable | No | No | No |
| 2 | Wada, Akihiko (2025) | No | Not applicable | No | Not applicable | No | No | No |
| 3 | Fink, Anna (2025) | No | Not applicable | Yes | Citation/source-file level | No | Yes | No |
| 4 | Kelly, Anthony (2025) | No | Not applicable | Yes | Citation/source/page level | No | Yes | No |
| 5 | Su, Audrey Y. (2025) | No | Not applicable | No | Not applicable | No | No | No |
| 6 | Chung Man Ho (2025) | No | Not applicable | No | Not applicable | No | No | No |
| 7 | Gan, Chunjing (2025) | Yes | Recall metrics; Normalized discounted cumulative gain (nDCG) | No | Not applicable | No | No | No |
| 8 | Tarabanis, Constantine (2026) | No | Not applicable | No | Not applicable | No | No | No |
| 9 | de Jesus, Davi dos Reis (2025) | No | Not applicable | No | Not applicable | No | No | No |
| 10 | David Baur (2025) | Yes | Precision-based retrieval metrics | Yes | Answer/claim level by RAGAS | Yes | No | No |
| 11 | Steybe, David (2025) | No | Not applicable | No | Not applicable | No | No | No |
| 12 | Yu, Deshui (2025) | Yes | Precision-based retrieval metrics; Recall metrics | Yes | Passage / answer statement | Yes | No | No |
| 13 | Dingqiao Wang (2024) | No | Not applicable | No | Not applicable | No | No | No |
| 14 | Dongchen Li (2026) | No | Not applicable | No | Not applicable | No | No | No |
| 15 | Busch (2025) | No | Not applicable | No | Not applicable | No | No | No |
| 16 | Hyunjae Kim (2025) | Yes | Precision-based retrieval metrics; Recall metrics | Yes | Passage/reference/statement level | Yes | Yes | No |
| 17 | Jiwoong Sohn (2025) | No | Not applicable | No | Not applicable | No | No | No |
| 18 | Junde Wu (2025) | No | Not applicable | Yes | Citation / answer level | No | Yes | No |
| 19 | Justice Ou (2025) | Yes | Other or not reported | No | Not applicable | No | No | No |
| 20 | Karthik Soman (2024) | Yes | Recall metrics | No | Not applicable | No | No | No |
| 21 | Krzysztof Wołk (2025) | Yes | Precision-based retrieval metrics; Mean reciprocal rank (MRR); Normalized discounted cumulative gain (nDCG) | Yes | Claim | Yes | No | Yes |
| 22 | Jonggwon Park (2026) | No | Not applicable | No | Not applicable | No | No | No |
| 23 | Leon Garza (2025) | Yes | Precision-based retrieval metrics | No | Not applicable | No | No | No |
| 24 | Linus Stuhlmann (2025) | Yes | Precision-based retrieval metrics; Recall metrics | No | Not applicable | No | No | No |
| 25 | Liping Xiong (2025) | No | Not applicable | No | Not applicable | No | No | No |
| 26 | Liwen Sun (2025) | No | Not applicable | No | Not applicable | No | No | No |
| 27 | M. Berkan Sesen (2025) | No | Not applicable | No | Not applicable | No | No | No |
| 28 | Madeline K. Moureau (2025) | No | Not applicable | No | Not applicable | No | No | Yes |
| 29 | Matthew Lewis (2025) | Yes | Precision-based retrieval metrics; Recall metrics; Mean reciprocal rank (MRR) | Yes | Claim / answer level by RAGAS | Yes | No | No |
| 30 | Michael Welsh (2025) | No | Not applicable | Yes | Citation/source relevance | No | Yes | No |
| 31 | Mohammad Alkhalaf (2024) | No | Not applicable | Yes | Summary/source level | No | No | No |
| 32 | Mohammad Reza Rezaei (2025) | No | Not applicable | No | Not applicable | No | No | No |
| 33 | Namrye Son (2025) | Yes | Precision-based retrieval metrics | No | Not applicable | No | No | No |
| 34 | Nuran Abdullayev (2025) | No | Not applicable | No | Not applicable | No | No | No |
| 35 | Valan, P. V. P. (2025) | No | Not applicable | No | Not applicable | No | No | No |
| 36 | Peng Xia (2024) | No | Not applicable | No | Not applicable | No | No | No |
| 37 | Philip DiGiacomo (2025) | No | Not applicable | Yes | Generated content / retrieved chunks | No | No | No |
| 38 | Rong Chen (2025) | No | Not applicable | No | Not applicable | No | No | No |
| 39 | Sebastian Wind (2025) | Yes | Other or not reported | Yes | Answer / retrieved context | No | No | Yes |
| 40 | Sheng-Ming Kuo (2025) | No | Not applicable | No | Not applicable | No | No | No |
| 41 | Sichu Liang (2025) | No | Not applicable | No | Not applicable | No | No | No |
| 42 | Siyang Liu (2025) | No | Not applicable | Yes | Answer / retrieved information | No | No | No |
| 43 | Skatje Myers (2025) | No | Not applicable | No | Not applicable | No | No | No |
| 44 | Tasnimul Hassan (2025) | No | Not applicable | Yes | Claim / unsupported claim annotation | Yes | No | No |
| 45 | Tharun Sekar (2025) | No | Not applicable | No | Not applicable | No | No | No |
| 46 | Vijaya Parameswaran (2025) | No | Not applicable | No | Not applicable | No | No | No |
| 47 | Wenchuan Zhang (2026) | Yes | Recall metrics; Mean reciprocal rank (MRR); Normalized discounted cumulative gain (nDCG) | No | Not applicable | No | No | No |
| 48 | Xiaowei Xu (2025) | Yes | Precision-based retrieval metrics; Recall metrics | No | Not applicable | No | No | No |
| 49 | Xuanzhao Dong (2025) | No | Not applicable | No | Not applicable | No | No | No |
| 50 | Xuejiao Zhao (2025) | No | Not applicable | No | Not applicable | No | No | No |
| 51 | Xueren Ge (2025) | Yes | Precision-based retrieval metrics; Recall metrics; Mean reciprocal rank (MRR) | No | Not applicable | No | No | No |
| 52 | Yash Mali (2025) | No | Not applicable | No | Not applicable | No | No | No |
| 53 | Yi Yu (2025) | Yes | Other or not reported | No | Not applicable | No | No | No |
| 54 | Yu He Ke (2025) | No | Not applicable | No | Not applicable | No | No | Yes |
| 55 | Yuelyu Ji (2025) | No | Not applicable | No | Not applicable | No | No | No |
| 56 | Zhongzhen Huang (2024) | Yes | Recall metrics | No | Not applicable | No | No | No |
| 57 | Zifeng Wang (2025) | No | Not applicable | No | Not applicable | No | No | No |
| 58 | Ziyu Wang (2025) | No | Not applicable | No | Not applicable | No | No | No |
| 59 | Xu, Ruiyu (2024) | No | Not applicable | No | Not applicable | No | No | No |
| 60 | Luo, Ming-Jie (2024) | No | Not applicable | No | Not applicable | No | No | No |
| 61 | Zhang, Gongbo (2025) | No | Not applicable | No | Not applicable | No | No | Yes |
| 62 | Low, Yen Sia (2025) | No | Not applicable | Yes | Citation / evidence source | No | Yes | No |
| 63 | Ge, Jin (2025) | No | Not applicable | No | Not applicable | No | No | No |
| 64 | Zakka, Cyril (2024) | No | Not applicable | Yes | Citation/factuality level | Yes | Yes | No |
| 65 | Tozuka, Ryota (2025) | Yes | Recall metrics | No | Not applicable | No | No | No |
| 66 | Hewitt, Katherine J (2024) | No | Not applicable | No | Not applicable | No | No | No |
| 67 | Wang, Dingqiao (2025) | No | Not applicable | No | Not applicable | No | No | No |
| 68 | Xu, Yupeng (2025) | No | Not applicable | No | Not applicable | No | No | No |
| 69 | Tung, Joshua Yi Min (2025) | No | Not applicable | No | Not applicable | No | No | No |
| 70 | Zhang, Chi (2025) | Yes | Precision-based retrieval metrics; Recall metrics | Yes | Answer/claim level by RAGAS | Yes | No | Yes |
| 71 | Fanelli, Francesco (2025) | No | Not applicable | No | Not applicable | No | No | No |
| 72 | Masanneck, Lars (2025a) | No | Not applicable | Yes | Citation/source level | No | Yes | No |
| 73 | Qimin Yang (2025) | Yes | Precision-based retrieval metrics; Mean reciprocal rank (MRR) | No | Not applicable | No | No | No |
| 74 | Marius Vach (2025) | Yes | Recall metrics | No | Not applicable | No | No | No |
| 75 | Masanneck, Lars (2025b) | No | Not applicable | No | Not applicable | No | No | No |
| 76 | Yusuke Fukui (2025) | Yes | Recall metrics | No | Not applicable | No | No | No |
| 77 | Hangyu Sha (2025) | No | Not applicable | No | Not applicable | No | No | No |
| 78 | Owoyemi, Joshua (2025) | Yes | Other or not reported | No | Not applicable | No | No | No |
| 79 | Tata, Venkatesh (2025) | Yes | Precision-based retrieval metrics; Mean reciprocal rank (MRR) | No | Not applicable | No | No | No |
| 80 | Tayebi Arasteh, Soroosh (2025) | Yes | Other or not reported | Yes | Answer/source suitability | No | No | No |
| 81 | Das, Sudeshna (2025) | No | Not applicable | Yes | Summary/source text | No | No | No |
| 82 | Hai Li (2025) | No | Not applicable | Yes | Claim | Yes | No | No |
| 83 | Shin, Minjeong (2025) | No | Not applicable | Yes | Citation/reference | Yes | Yes | No |
| 84 | Aguzzi, Gianluca (2025) | No | Not applicable | Yes | Claim | Yes | No | No |
| 85 | Zhou, Qingqing (2024) | Yes | Recall metrics | Yes | Assertion / RAGAS | Yes | No | No |
| 86 | Aminan, Mohammad (2025) | Yes | Precision-based retrieval metrics; Recall metrics | Yes | Claim / RAGAS | Yes | No | No |
| 87 | Hsu, Hsin-Ling (2025) | No | Not applicable | No | Not applicable | No | No | No |
| 88 | Kresevic, Simone (2024) | No | Not applicable | No | Not applicable | No | No | No |
| 89 | Kang, Bongsu (2024) | No | Not applicable | No | Not applicable | No | No | No |
| 90 | AlSammarraie, AlHasan (2025) | No | Not applicable | No | Not applicable | No | No | No |
| 91 | Nandy, Gargi (2025) | No | Not applicable | No | Not applicable | No | No | No |
| 92 | Hetz, M. J. (2024) | No | Not applicable | No | Not applicable | No | No | No |
| 93 | Ong, Chin Siang (2024) | No | Not applicable | No | Not applicable | No | No | No |
| 94 | Nicolas Carl (2026) | No | Not applicable | Yes | Citation/source / recommendation alignment | No | Yes | No |
| 95 | Tytler, Kolawole (2025) | No | Not applicable | No | Not applicable | No | No | No |
| 96 | Wu, Yue (2025) | No | Not applicable | No | Not applicable | No | No | No |
| 97 | Kim, Seonok (2025) | Yes | Precision-based retrieval metrics; Recall metrics; Mean reciprocal rank (MRR); Normalized discounted cumulative gain (nDCG) | No | Not applicable | No | No | No |
| 98 | Hasan, Md. Mehedi (2025) | Yes | Precision-based retrieval metrics | No | Not applicable | No | No | No |
| 99 | Long, Yongchao (2025) | No | Not applicable | No | Not applicable | No | No | No |
| 100 | Zhang, Jinning (2026) | Yes | Recall metrics; Normalized discounted cumulative gain (nDCG) | Yes | Claim / retrieved context | Yes | No | No |
| 101 | Ryan, Jessica (2026) | No | Not applicable | No | Not applicable | No | No | No |
| 102 | Yang, Weiqin (2026) | No | Not applicable | No | Not applicable | No | No | No |
| 103 | Lorenzo, Luis (2026) | No | Not applicable | No | Not applicable | No | No | No |
| 104 | Li, Zheng (2026) | No | Not applicable | No | Not applicable | No | No | No |
| 105 | Samanta, Himadri (2026) | Yes | Recall metrics | No | Not applicable | No | No | No |
| 106 | Chan, Richard Wai Cheung (2026) | No | Not applicable | No | Not applicable | No | No | No |
| 107 | Chen, Xupeng (2026) | Yes | Recall metrics | No | Not applicable | No | No | No |
| 108 | Khosa, Thokozile (2026) | Yes | Recall metrics | No | Not applicable | No | No | No |
| 109 | Abdul Rehman Akbar (2026) | Yes | Recall metrics | Yes | Fact / answer | Yes | No | No |
| 110 | Jianmin Li (2026) | Yes | Precision-based retrieval metrics; Recall metrics | No | Not applicable | No | No | No |
| 111 | Haoqing Li (2025) | No | Not applicable | No | Not applicable | No | No | No |
| 112 | Zhe Chen (2025b) | No | Not applicable | No | Not applicable | No | No | No |
| 113 | Wenhao Li (2025) | Yes | Precision-based retrieval metrics; Mean reciprocal rank (MRR) | No | Not applicable | No | No | No |
| 114 | Mohammad Shoaib Ansari (2025) | No | Not applicable | No | Not applicable | No | No | No |
| 115 | Peng Xia (2025) | Yes | Recall metrics | No | Not applicable | No | No | No |
| 116 | Yingpeng Ning (2025) | No | Not applicable | Yes | Statement/citation | Yes | Yes | No |
| 117 | Houman Kazemzadeh (2025) | No | Not applicable | No | Not applicable | No | No | No |
| 118 | Yingjian Chen (2025) | No | Not applicable | No | Not applicable | No | No | No |
| 119 | Han, Zihan (2025) | No | Not applicable | No | Not applicable | No | No | No |
| 120 | Garapati, Keerthana (2025) | No | Not applicable | No | Not applicable | No | No | No |
| 121 | Guo, Zhijun (2025) | No | Not applicable | No | Not applicable | No | No | No |
| 122 | Zhu, Yu (2026) | No | Not applicable | No | Not applicable | Not applicable | No | Yes |
| 123 | Mo, Gao (2026) | Yes | Other or not reported | No | Not applicable | No | No | No |
| 124 | Shahnawaz, Amna (2026) | No | Not applicable | No | Not applicable | No | No | No |
| 125 | Ahalpara, Trishala Jayesh (2025) | No | Not applicable | Yes | Answer level | No | No | No |
| 126 | Boumans, Roel (2025) | No | Not applicable | No | Not applicable | No | No | No |
| 127 | Parmanto, Bambang (2024) | No | Not applicable | Yes | Citation | Yes | Yes | No |
| 128 | Hang, Ching Nam (2025) | No | Not applicable | Yes | Claim | Yes | No | Yes |
| 129 | Gu, Difei (2025) | No | Not applicable | No | Not applicable | No | No | No |
| 130 | Yi, Ziruo (2025) | No | Not applicable | No | Not applicable | No | No | No |
| 131 | Bang, Byeonghun (2026) | Yes | Other or not reported | Yes | Rationale / answer-level evidence attribution | Yes | No | No |
| 132 | Ting, Lo Pang-Yun (2025) | No | Not applicable | No | Not applicable | No | No | No |
| 133 | Johno, Hisashi (2026) | Yes | Recall metrics | Yes | Claim | Yes | No | Yes |
| 134 | He, Jinglin (2025) | No | Not applicable | No | Not applicable | No | No | No |
| 135 | Zhao, Yi-Fei (2025) | No | Not applicable | No | Not applicable | No | No | No |
| 136 | Madrid-García, Alfredo (2025) | No | Not applicable | Yes | Answer-level retrieved-context faithfulness | No | No | No |
| 137 | Saidu, Fatima (2026) | Yes | Precision-based retrieval metrics; Recall metrics | Yes | Answer-level / RAGAS | No | No | No |
| 138 | Felde, Sabine (2026) | No | Not applicable | Yes | RAGAS factual consistency/relevance | No | No | No |
| 139 | Jeon, Yaejin (2026) | Yes | Precision-based retrieval metrics; Recall metrics | Yes | Claim / RAGAS | Yes | No | No |
| 140 | Kang et al. (2026) | No | Not applicable | No | Not applicable | No | No | No |
| 141 | Komenda et al. (2026) | Yes | Recall metrics | No | Not applicable | No | No | No |
| 142 | Wang et al. (2026) | No | Not applicable | No | Not applicable | No | No | No |
| 143 | Zhang et al. (2026) | Yes | Recall metrics; Normalized discounted cumulative gain (nDCG) | Yes | Claim | Yes | No | No |
| 144 | Saadi, Soroush Baseri (2026) | No | Not applicable | No | Not applicable | No | No | No |
| 145 | Song, Jong Keon (2026) | No | Not applicable | No | Not applicable | No | No | No |
| 146 | Kabak, Yildiray (2025) | Yes | Precision-based retrieval metrics; Recall metrics | Yes | Answer-level / RAGAS | No | No | No |
| 147 | Ma, Jiaqing (2026) | No | Not applicable | No | Not applicable | No | No | No |
| 148 | Anonymous ACL submission (2026) | Yes | Precision-based retrieval metrics; Recall metrics | No | Not applicable | No | No | No |
| 149 | Garcia-Font, Marc (2026) | No | Not applicable | No | Not applicable | No | No | No |
| 150 | Wong, Hang Sheung (2026) | No | Not applicable | No | Not applicable | No | No | No |
| 151 | Thio et al. (2026) | Yes | Precision-based retrieval metrics; Recall metrics | Yes | Source-note factual support | No | No | No |
| 152 | Zaki et al. (2026) | No | Not applicable | No | Not applicable | No | No | No |
| 153 | Liu, Haixiao (2026) | No | Not applicable | No | Not applicable | No | No | No |
| 154 | Kim, DaeHo (2026) | No | Not applicable | No | Not applicable | No | No | No |
| 155 | Lopez, Ivan (2025) | No | Not applicable | No | Not applicable | No | No | No |
| 156 | Nanua, Suparna (2025) | No | Not applicable | No | Not applicable | No | No | No |
| 157 | Xie, Wenbo (2026) | No | Not applicable | No | Not applicable | No | No | No |

**Table S3. Evaluation modalities, safety-related evaluation, GraphRAG-specific indicators, and governance reporting**

| **Study No.** | **Citation** | **Human evaluation** | **Interrater reliability reported** | **Automated metrics reported** | **LLM-as-judge evaluation** | **LLM-as-judge bias-control measures** | **Combined human and LLM evaluation** | **Formal safety-related evaluation** | **GraphRAG minimum criterion** | **GraphRAG construction evaluation** | **GraphRAG intermediate-artifact evaluation** | **Use of real patient data** | **De-identification reported** | **IRB approval, exemption, or waiver reported** |
| --- | --- | --- | --- | --- | --- | --- | --- | --- | --- | --- | --- | --- | --- | --- |
| 1 | Karim, A H M Rezaul (2025) | No | Not reported | Yes | Yes | No | No | No | No | No | No | No | Not reported | Not reported |
| 2 | Wada, Akihiko (2025) | Yes | Yes | No | Yes | Yes | Yes | Yes | No | No | No | No | Not reported | Yes |
| 3 | Fink, Anna (2025) | Yes | Yes | No | No | Not applicable | No | No | No | No | No | No | Not reported | Yes |
| 4 | Kelly, Anthony (2025) | Yes | Not reported | No | No | Not applicable | No | No | No | No | No | No | Not reported | Yes |
| 5 | Su, Audrey Y. (2025) | Yes | No | No | No | Not applicable | No | No | No | No | No | No | Not reported | Yes |
| 6 | Chung Man Ho (2025) | Yes | Yes | No | No | Not applicable | No | No | No | No | No | No | Yes | Yes |
| 7 | Gan, Chunjing (2025) | No | Not reported | Yes | Yes | No | No | No | No | No | No | No | Not reported | Not reported |
| 8 | Tarabanis, Constantine (2026) | No | Not reported | Yes | No | Not applicable | No | No | No | No | No | No | Not reported | Yes |
| 9 | de Jesus, Davi dos Reis (2025) | Yes | Not reported | No | No | Not applicable | No | Yes | No | No | No | No | Yes | Yes |
| 10 | David Baur (2025) | Yes | Not reported | No | Yes | Not reported | Yes | No | No | No | No | No | Not reported | Yes |
| 11 | Steybe, David (2025) | Yes | Not reported | No | No | Not applicable | No | No | No | No | No | No | Not reported | Not reported |
| 12 | Yu, Deshui (2025) | No | Not reported | Yes | Yes | Not reported | No | No | No | No | No | No | Not reported | Not reported |
| 13 | Dingqiao Wang (2024) | Yes | Not reported | No | No | Not applicable | No | No | No | No | No | No | Not reported | Yes |
| 14 | Dongchen Li (2026) | Yes | Not reported | Yes | Yes | Not reported | Yes | Yes | Yes | No | No | Yes | Not reported | Not reported |
| 15 | Busch (2025) | Yes | Not reported | No | No | Not applicable | No | No | No | No | No | Yes | Yes | Yes |
| 16 | Hyunjae Kim (2025) | Yes | Yes | Yes | No | Not applicable | No | No | No | No | No | No | Not reported | Not reported |
| 17 | Jiwoong Sohn (2025) | No | Not reported | Yes | No | Not applicable | No | No | No | No | No | No | Not reported | Not reported |
| 18 | Junde Wu (2025) | Yes | Not reported | Yes | No | Not applicable | No | Yes | Yes | Yes | Yes | Yes | Not reported | Not reported |
| 19 | Justice Ou (2025) | No | Not reported | Yes | Yes | Not reported | No | No | No | No | No | Yes | Yes | Yes |
| 20 | Karthik Soman (2024) | No | Not reported | Yes | No | Not applicable | No | No | Yes | No | Yes | No | Not reported | Not reported |
| 21 | Krzysztof Wołk (2025) | Yes | Yes | Yes | No | Not applicable | No | Yes | Yes | No | No | Yes | Yes | Not reported |
| 22 | Jonggwon Park (2026) | No | Not applicable | Yes | No | Not applicable | No | No | No | Not applicable | Not applicable | Yes | Not reported | Not reported |
| 23 | Leon Garza (2025) | No | Not applicable | Yes | No | Not applicable | No | No | No | Not applicable | Not applicable | Yes | Yes | Yes |
| 24 | Linus Stuhlmann (2025) | No | Not applicable | Yes | No | Not applicable | No | No | No | Not applicable | Not applicable | No | Not applicable | Not applicable |
| 25 | Liping Xiong (2025) | Yes | Not applicable | Yes | No | Not applicable | No | Yes | No | Not applicable | Not applicable | Yes | Yes | Yes |
| 26 | Liwen Sun (2025) | No | Not applicable | Yes | No | Not applicable | No | No | No | Not applicable | Not applicable | Yes | Not reported | Not reported |
| 27 | M. Berkan Sesen (2025) | Yes | Not applicable | No | Yes | Not reported | Yes | No | Yes | No | No | No | Not applicable | Not applicable |
| 28 | Madeline K. Moureau (2025) | Yes | Yes | No | No | Not applicable | No | No | No | Not applicable | Not applicable | No | Not applicable | Yes |
| 29 | Matthew Lewis (2025) | Yes | Not applicable | Yes | Yes | Yes | Yes | Yes | No | Not applicable | Not applicable | No | Not applicable | Yes |
| 30 | Michael Welsh (2025) | Yes | No | No | No | Not applicable | No | No | No | Not applicable | Not applicable | No | Not applicable | Yes |
| 31 | Mohammad Alkhalaf (2024) | Yes | Not reported | Yes | No | Not applicable | No | No | No | Not applicable | Not applicable | Yes | Yes | Yes |
| 32 | Mohammad Reza Rezaei (2025) | No | Not applicable | Yes | Yes | Not applicable | No | No | Yes | Yes | Yes | No | Not applicable | Not reported |
| 33 | Namrye Son (2025) | No | Not applicable | Yes | No | Not applicable | No | No | No | Not applicable | Not applicable | No | Not applicable | Not reported |
| 34 | Nuran Abdullayev (2025) | No | Not applicable | No | No | Not applicable | No | No | No | Not applicable | Not applicable | Yes | Yes | Yes |
| 35 | Valan, P. V. P. (2025) | Yes | No | No | No | Not applicable | No | No | No | Not applicable | Not applicable | No | Not applicable | Yes |
| 36 | Peng Xia (2024) | No | Not applicable | Yes | No | Not applicable | No | No | No | Not applicable | Not applicable | Yes | Not reported | Not reported |
| 37 | Philip DiGiacomo (2025) | No | Not applicable | No | Yes | Yes | No | No | No | Not applicable | Not applicable | No | Not applicable | Not applicable |
| 38 | Rong Chen (2025) | Yes | No | No | No | Not applicable | No | No | No | Not applicable | Not applicable | No | Not reported | Not reported |
| 39 | Sebastian Wind (2025) | Yes | Not applicable | Yes | Yes | Not applicable | Yes | Yes | No | Not applicable | Not applicable | No | Not applicable | Yes |
| 40 | Sheng-Ming Kuo (2025) | Yes | Yes | Yes | No | Not applicable | No | Yes | No | Not applicable | Not applicable | Yes | Yes | Yes |
| 41 | Sichu Liang (2025) | No | Not applicable | Yes | No | Not applicable | No | No | No | Not applicable | Not applicable | Yes | Not reported | Not reported |
| 42 | Siyang Liu (2025) | Yes | Not reported | No | No | Not applicable | No | No | No | Not applicable | Not applicable | No | Not applicable | Yes |
| 43 | Skatje Myers (2025) | No | Not applicable | Yes | No | Not applicable | No | No | No | Not applicable | Not applicable | Yes | Not reported | Not reported |
| 44 | Tasnimul Hassan (2025) | Yes | Yes | Yes | No | Not applicable | No | No | No | Not applicable | Not applicable | No | Not applicable | Not applicable |
| 45 | Tharun Sekar (2025) | No | Not applicable | Yes | No | Not applicable | No | No | Yes | No | No | No | Not applicable | Not applicable |
| 46 | Vijaya Parameswaran (2025) | Yes | Yes | No | No | Not applicable | No | Yes | No | Not applicable | Not applicable | No | Not applicable | Yes |
| 47 | Wenchuan Zhang (2026) | No | Not applicable | Yes | No | Not applicable | No | No | No | Not applicable | Not applicable | No | Not applicable | Not applicable |
| 48 | Xiaowei Xu (2025) | Yes | No | No | No | Not applicable | No | No | No | Not applicable | Not applicable | No | Not applicable | Yes |
| 49 | Xuanzhao Dong (2025) | No | Not applicable | Yes | No | Not applicable | No | No | No | Not applicable | Not applicable | No | Not applicable | Not applicable |
| 50 | Xuejiao Zhao (2025) | No | Not applicable | Yes | No | Not applicable | No | No | Yes | No | No | Yes | Not reported | Not reported |
| 51 | Xueren Ge (2025) | No | Not applicable | Yes | No | Not applicable | No | No | No | Not applicable | Not applicable | No | Not reported | Not reported |
| 52 | Yash Mali (2025) | Yes | Not reported | No | No | Not applicable | No | Yes | No | Not applicable | Not applicable | No | Not applicable | Not applicable |
| 53 | Yi Yu (2025) | Yes | Yes | No | No | Not applicable | No | No | No | Not applicable | Not applicable | No | Not applicable | Not applicable |
| 54 | Yu He Ke (2025) | Yes | No | Yes | Yes | Not applicable | Yes | Yes | No | Not applicable | Not applicable | Yes | Yes | Yes |
| 55 | Yuelyu Ji (2025) | No | Not applicable | Yes | No | Not applicable | No | No | No | Not applicable | Not applicable | No | Not applicable | Not applicable |
| 56 | Zhongzhen Huang (2024) | No | Not applicable | Yes | No | Not applicable | No | No | No | Not applicable | Not applicable | No | Not applicable | Not applicable |
| 57 | Zifeng Wang (2025) | Yes | Not reported | Yes | Yes | Yes | Yes | No | No | Not applicable | Not applicable | No | Not applicable | Not reported |
| 58 | Ziyu Wang (2025) | No | Not applicable | Yes | No | Not applicable | No | No | No | Not applicable | Not applicable | No | Not applicable | Not applicable |
| 59 | Xu, Ruiyu (2024) | Yes | No | No | No | Not applicable | No | No | No | Not applicable | Not applicable | No | Not applicable | Yes |
| 60 | Luo, Ming-Jie (2024) | Yes | Not reported | Yes | No | Not applicable | No | Yes | No | Not applicable | Not applicable | Yes | Yes | Yes |
| 61 | Zhang, Gongbo (2025) | Yes | No | Yes | No | Not applicable | No | No | No | Not applicable | Not applicable | No | Not applicable | Not applicable |
| 62 | Low, Yen Sia (2025) | Yes | Yes | No | No | Not applicable | No | No | No | Not applicable | Not applicable | Yes | Yes | Yes |
| 63 | Ge, Jin (2025) | Yes | Not reported | No | No | Not applicable | No | Yes | No | Not applicable | Not applicable | No | Not applicable | Yes |
| 64 | Zakka, Cyril (2024) | Yes | Yes | No | No | Not applicable | No | Yes | No | Not applicable | Not applicable | No | Not applicable | Not applicable |
| 65 | Tozuka, Ryota (2025) | Yes | No | Yes | No | Not applicable | No | No | No | Not applicable | Not applicable | No | Not applicable | Not applicable |
| 66 | Hewitt, Katherine J (2024) | Yes | Not applicable | No | No | Not applicable | No | No | No | Not applicable | Not applicable | Yes | Yes | Not applicable |
| 67 | Wang, Dingqiao (2025) | Yes | No | Yes | No | Not applicable | No | No | No | Not applicable | Not applicable | No | Not applicable | Yes |
| 68 | Xu, Yupeng (2025) | Yes | No | Yes | No | Not applicable | No | Yes | No | Not applicable | Not applicable | Yes | Yes | Yes |
| 69 | Tung, Joshua Yi Min (2025) | No | Not applicable | No | No | Not applicable | No | No | No | Not applicable | Not applicable | No | Not applicable | Yes |
| 70 | Zhang, Chi (2025) | Yes | No | Yes | Yes | No | Yes | Yes | No | Not applicable | Not applicable | Yes | Yes | Yes |
| 71 | Fanelli, Francesco (2025) | Yes | Yes | Yes | No | Not applicable | No | No | No | Not applicable | Not applicable | No | Not applicable | Not applicable |
| 72 | Masanneck, Lars (2025a) | Yes | Yes | No | No | Not applicable | No | Yes | No | Not applicable | Not applicable | No | Not applicable | Not applicable |
| 73 | Qimin Yang (2025) | Yes | No | No | No | Not applicable | No | No | No | Not applicable | Not applicable | No | Not reported | Not reported |
| 74 | Marius Vach (2025) | Yes | No | Yes | No | Not applicable | No | Yes | No | Not applicable | Not applicable | No | Not applicable | Yes |
| 75 | Masanneck, Lars (2025b) | Yes | Yes | No | No | Not applicable | No | No | No | Not applicable | Not applicable | No | Not applicable | Not applicable |
| 76 | Yusuke Fukui (2025) | Yes | Yes | Yes | Yes | No | Yes | No | No | Not applicable | Not applicable | No | Not applicable | Yes |
| 77 | Hangyu Sha (2025) | Yes | No | Yes | No | Not applicable | No | No | Yes | No | No | No | Not applicable | Yes |
| 78 | Owoyemi, Joshua (2025) | Yes | No | Yes | No | Not applicable | No | No | No | Not applicable | Not applicable | No | Not applicable | Not reported |
| 79 | Tata, Venkatesh (2025) | Yes | No | Yes | No | Not applicable | No | No | Yes | No | Yes | No | Not applicable | Not reported |
| 80 | Tayebi Arasteh, Soroosh (2025) | Yes | No | Yes | No | Not applicable | No | Yes | No | Not applicable | Not applicable | No | Not applicable | Yes |
| 81 | Das, Sudeshna (2025) | Yes | No | Yes | No | Not applicable | No | No | No | Not applicable | Not applicable | No | Yes | Yes |
| 82 | Hai Li (2025) | No | Not applicable | Yes | Yes | Not applicable | No | Yes | No | Not applicable | Not applicable | No | Not applicable | Yes |
| 83 | Shin, Minjeong (2025) | Yes | No | No | No | Not applicable | No | Yes | No | Not applicable | Not applicable | No | Not applicable | Yes |
| 84 | Aguzzi, Gianluca (2025) | Yes | No | Yes | Yes | Yes | Yes | Yes | No | Not applicable | Not applicable | No | Not applicable | Yes |
| 85 | Zhou, Qingqing (2024) | Yes | No | Yes | Yes | No | Yes | Yes | No | Not applicable | Not applicable | No | Not applicable | Not applicable |
| 86 | Aminan, Mohammad (2025) | No | Not applicable | Yes | Yes | Not reported | No | No | No | Not applicable | Not applicable | Yes | Yes | Not reported |
| 87 | Hsu, Hsin-Ling (2025) | No | No | Yes | No | Not applicable | No | No | No | Not applicable | Not applicable | Yes | Yes | Not reported |
| 88 | Kresevic, Simone (2024) | Yes | No | Yes | No | Not applicable | No | No | No | Not applicable | Not applicable | No | Not applicable | Not applicable |
| 89 | Kang, Bongsu (2024) | Yes | No | No | No | Not applicable | No | No | No | Not applicable | Not applicable | No | Not applicable | Not applicable |
| 90 | AlSammarraie, AlHasan (2025) | Yes | No | Yes | Yes | Yes | Yes | Yes | No | Not applicable | Not applicable | No | Not applicable | Yes |
| 91 | Nandy, Gargi (2025) | Yes | No | No | No | Not applicable | No | No | No | Not applicable | Not applicable | No | Not reported | Yes |
| 92 | Hetz, M. J. (2024) | No | Not applicable | Yes | No | Not applicable | No | No | No | Not applicable | Not applicable | No | Not applicable | Not applicable |
| 93 | Ong, Chin Siang (2024) | No | Not applicable | No | No | Not applicable | No | No | No | Not applicable | Not applicable | No | Not applicable | Not applicable |
| 94 | Nicolas Carl (2026) | Yes | Yes | No | No | Not applicable | No | No | No | Not applicable | Not applicable | No | Not reported | Yes |
| 95 | Tytler, Kolawole (2025) | Yes | No | No | No | Not applicable | No | No | No | Not applicable | Not applicable | No | Yes | Yes |
| 96 | Wu, Yue (2025) | Yes | No | Yes | No | Not applicable | No | Yes | No | Not applicable | Not applicable | Yes | Yes | Yes |
| 97 | Kim, Seonok (2025) | No | Not applicable | Yes | No | Not applicable | No | No | No | Not applicable | Not applicable | No | Not applicable | Not applicable |
| 98 | Hasan, Md. Mehedi (2025) | Yes | Not reported | Yes | No | Not applicable | No | Yes | No | Not applicable | Not applicable | No | Not applicable | Not applicable |
| 99 | Long, Yongchao (2025) | No | Not applicable | Yes | No | Not applicable | No | No | No | Not applicable | Not applicable | No | Not applicable | Not applicable |
| 100 | Zhang, Jinning (2026) | Yes | Yes | Yes | Yes | Yes | Yes | Yes | Yes | No | Yes | No | Not applicable | Not applicable |
| 101 | Ryan, Jessica (2026) | No | Not applicable | Yes | No | Not applicable | No | No | No | Not applicable | Not applicable | No | Not applicable | Not applicable |
| 102 | Yang, Weiqin (2026) | No | Not applicable | Yes | No | Not applicable | No | No | Yes | No | No | Yes | Not reported | Not applicable |
| 103 | Lorenzo, Luis (2026) | No | Not applicable | Yes | No | Not applicable | No | No | No | Not applicable | Not applicable | No | Not applicable | Not applicable |
| 104 | Li, Zheng (2026) | No | Not applicable | Yes | No | Not applicable | No | No | Yes | No | No | Yes | Yes | Not reported |
| 105 | Samanta, Himadri (2026) | No | Not applicable | Yes | No | Not applicable | No | Yes | No | Not applicable | Not applicable | Yes | Yes | Not reported |
| 106 | Chan, Richard Wai Cheung (2026) | No | Not applicable | Yes | Yes | Not applicable | No | No | No | Not applicable | Not applicable | No | Not applicable | Not applicable |
| 107 | Chen, Xupeng (2026) | No | Not applicable | Yes | Yes | Not applicable | No | No | No | Not applicable | Not applicable | No | Not applicable | Not applicable |
| 108 | Khosa, Thokozile (2026) | No | Not applicable | Yes | Yes | Not applicable | No | No | Yes | No | Yes | No | Not applicable | Not applicable |
| 109 | Abdul Rehman Akbar (2026) | Yes | No | Yes | No | Not applicable | No | No | No | Not applicable | Not applicable | Yes | Yes | Yes |
| 110 | Jianmin Li (2026) | No | Not applicable | Yes | Yes | Not applicable | No | No | Yes | No | Yes | No | Not reported | Not reported |
| 111 | Haoqing Li (2025) | Yes | Not applicable | Yes | No | Not applicable | No | No | No | Not applicable | Not applicable | Yes | Not reported | Not reported |
| 112 | Zhe Chen (2025b) | No | Not applicable | Yes | Yes | Not applicable | No | No | Yes | Not applicable | Not applicable | Yes | Not reported | Not reported |
| 113 | Wenhao Li (2025) | No | Not applicable | Yes | No | Not applicable | No | No | No | Not applicable | Not applicable | Yes | Yes | Yes |
| 114 | Mohammad Shoaib Ansari (2025) | No | Not applicable | Yes | No | Not applicable | No | No | No | Not applicable | Not applicable | No | Not reported | Not reported |
| 115 | Peng Xia (2025) | No | Not applicable | Yes | No | Not applicable | No | No | No | Not applicable | Not applicable | Yes | Yes | Not reported |
| 116 | Yingpeng Ning (2025) | No | Not applicable | Yes | Yes | No | No | Yes | No | Not applicable | Not applicable | No | Not applicable | Not applicable |
| 117 | Houman Kazemzadeh (2025) | No | Not applicable | Yes | No | Not applicable | No | No | No | Not applicable | Not applicable | No | Not applicable | Not applicable |
| 118 | Yingjian Chen (2025) | No | Not applicable | Yes | No | Not applicable | No | No | Yes | No | No | No | Not applicable | Not applicable |
| 119 | Han, Zihan (2025) | No | Not applicable | Yes | No | Not applicable | No | Yes | Yes | No | No | Yes | Yes | Not reported |
| 120 | Garapati, Keerthana (2025) | No | Not applicable | Yes | Yes | Yes | No | No | No | Not applicable | Not applicable | Yes | Yes | Not applicable |
| 121 | Guo, Zhijun (2025) | Yes | Not applicable | Yes | No | Not applicable | No | Yes | No | Not applicable | Not applicable | No | Yes | Yes |
| 122 | Zhu, Yu (2026) | No | Not applicable | Yes | No | Not applicable | No | No | No | Not applicable | Not applicable | Yes | Not reported | Yes |
| 123 | Mo, Gao (2026) | Yes | No | No | Yes | Yes | Yes | No | No | Not applicable | Not applicable | No | Yes | Yes |
| 124 | Shahnawaz, Amna (2026) | Yes | No | No | No | Not applicable | No | Yes | No | Not applicable | Not applicable | No | Not applicable | Yes |
| 125 | Ahalpara, Trishala Jayesh (2025) | Yes | Not applicable | Yes | Yes | Yes | Yes | Yes | No | Not applicable | Not applicable | No | Not applicable | Not reported |
| 126 | Boumans, Roel (2025) | Yes | Not applicable | No | No | Not applicable | No | No | No | Not applicable | Not applicable | No | Not applicable | Yes |
| 127 | Parmanto, Bambang (2024) | Yes | Not applicable | Yes | No | Not applicable | No | No | No | Not applicable | Not applicable | No | Yes | Yes |
| 128 | Hang, Ching Nam (2025) | No | Not applicable | Yes | No | Not applicable | No | Yes | Yes | Yes | Yes | No | Not applicable | Not applicable |
| 129 | Gu, Difei (2025) | No | Not applicable | Yes | Yes | Not reported | No | No | No | Not applicable | Not applicable | Yes | Not reported | Not reported |
| 130 | Yi, Ziruo (2025) | No | Not applicable | Yes | Yes | Not reported | No | No | No | Not applicable | Not applicable | Yes | Not reported | Not applicable |
| 131 | Bang, Byeonghun (2026) | No | Not reported | Yes | No | Not applicable | No | Yes | No | No | No | No | Not applicable | Not applicable |
| 132 | Ting, Lo Pang-Yun (2025) | Yes | No | Yes | No | Not applicable | No | No | Yes | No | No | Yes | Yes | Not applicable |
| 133 | Johno, Hisashi (2026) | Yes | No | Yes | No | Not applicable | No | No | No | Not applicable | Not applicable | No | Not applicable | Not applicable |
| 134 | He, Jinglin (2025) | Yes | Yes | Yes | No | Not applicable | No | No | Yes | Yes | No | No | Not applicable | Not reported |
| 135 | Zhao, Yi-Fei (2025) | Yes | Yes | Yes | No | Not applicable | No | No | No | Not applicable | Not applicable | No | Not applicable | Not applicable |
| 136 | Madrid-García, Alfredo (2025) | Yes | Yes | Yes | Yes | Yes | Yes | Yes | No | Not applicable | Not applicable | No | Not applicable | Yes |
| 137 | Saidu, Fatima (2026) | Yes | Not applicable | Yes | Yes | Not applicable | Yes | No | Yes | No | Yes | No | Not applicable | Yes |
| 138 | Felde, Sabine (2026) | Yes | No | Yes | Yes | Not reported | Yes | No | No | Not applicable | Not applicable | Yes | Yes | Not reported |
| 139 | Jeon, Yaejin (2026) | Yes | Yes | Yes | Yes | Yes | Yes | No | No | Not applicable | Not applicable | No | Not applicable | Yes |
| 140 | Kang et al. (2026) | Yes | Yes | Yes | Yes | Yes | Yes | Yes | No | Not applicable | Not applicable | Yes | Yes | Yes |
| 141 | Komenda et al. (2026) | Yes | No | Yes | No | Not applicable | No | No | No | Not applicable | Not applicable | No | Not applicable | Yes |
| 142 | Wang et al. (2026) | Yes | Yes | Yes | No | Not applicable | No | Yes | No | Not applicable | Not applicable | No | Not applicable | Yes |
| 143 | Zhang et al. (2026) | Yes | Yes | Yes | Yes | Yes | Yes | Yes | Yes | Yes | Yes | No | Not applicable | Not reported |
| 144 | Saadi, Soroush Baseri (2026) | No | Not reported | Yes | No | Not applicable | No | No | No | Not applicable | Not applicable | Yes | Yes | Yes |
| 145 | Song, Jong Keon (2026) | Yes | No | Yes | Yes | Yes | Yes | No | Yes | Yes | No | No | Not applicable | Not reported |
| 146 | Kabak, Yildiray (2025) | Yes | Not reported | Yes | Yes | Not reported | Yes | No | No | Not applicable | Not applicable | No | Not reported | Yes |
| 147 | Ma, Jiaqing (2026) | No | Not applicable | Yes | No | Not applicable | No | No | Yes | No | No | Yes | Not reported | Not reported |
| 148 | Anonymous ACL submission (2026) | Yes | No | Yes | No | Not applicable | No | No | No | Not applicable | Not applicable | Yes | Not reported | Not reported |
| 149 | Garcia-Font, Marc (2026) | Yes | No | Yes | No | Not applicable | No | No | No | Not applicable | Not applicable | No | Not applicable | Yes |
| 150 | Wong, Hang Sheung (2026) | No | Not applicable | Yes | No | Not applicable | No | Yes | No | Not applicable | Not applicable | Yes | Yes | Yes |
| 151 | Thio et al. (2026) | Yes | No | Yes | No | Not applicable | No | Yes | Yes | No | Yes | Yes | Yes | Yes |
| 152 | Zaki et al. (2026) | Yes | No | Yes | No | Not applicable | No | No | No | Not applicable | Not applicable | Yes | Yes | Yes |
| 153 | Liu, Haixiao (2026) | No | Not applicable | Yes | No | Not applicable | No | Yes | No | No | Not applicable | Yes | Yes | Yes |
| 154 | Kim, DaeHo (2026) | No | Not reported | Yes | No | Not applicable | No | No | Yes | No | No | No | Not applicable | Not applicable |
| 155 | Lopez, Ivan (2025) | No | Not applicable | Yes | No | Not applicable | No | No | No | Not applicable | Not applicable | Yes | Yes | Yes |
| 156 | Nanua, Suparna (2025) | Yes | No | Yes | No | Not applicable | No | Yes | No | Not applicable | Not applicable | No | Not applicable | Not applicable |
| 157 | Xie, Wenbo (2026) | Yes | Yes | Yes | No | Not applicable | No | Yes | No | Not applicable | Not applicable | No | Yes | Yes |

**Table S4. Source data for Figure 3**

| **Figure** | **Panel or evaluation domain** | **Item or evaluation-setting category** | **Numerator or study count** | **Denominator** | **Percentage or within-category coverage proportion** |
| --- | --- | --- | --- | --- | --- |
| Figure 3 | Retrieval-layer evaluation | Offline-only evaluation | 42 | 140 | 42/140 (30.0%) |
| Figure 3 | Retrieval-layer evaluation | Simulated vignette or case evaluation | 12 | 37 | 12/37 (32.4%) |
| Figure 3 | Retrieval-layer evaluation | Workflow pilot or user study | 5 | 17 | 5/17 (29.4%) |
| Figure 3 | Retrieval-layer evaluation | Prospective clinical study | 0 | 2 | 0/2 (0.0%) |
| Figure 3 | Retrieval-layer evaluation | Real-world deployment or postdeployment monitoring | 1 | 3 | 1/3 (33.3%) |
| Figure 3 | Fine-grained evidence verification | Offline-only evaluation | 20 | 140 | 20/140 (14.3%) |
| Figure 3 | Fine-grained evidence verification | Simulated vignette or case evaluation | 5 | 37 | 5/37 (13.5%) |
| Figure 3 | Fine-grained evidence verification | Workflow pilot or user study | 2 | 17 | 2/17 (11.8%) |
| Figure 3 | Fine-grained evidence verification | Prospective clinical study | 0 | 2 | 0/2 (0.0%) |
| Figure 3 | Fine-grained evidence verification | Real-world deployment or postdeployment monitoring | 1 | 3 | 1/3 (33.3%) |
| Figure 3 | Citation and source correctness evaluation | Offline-only evaluation | 11 | 140 | 11/140 (7.9%) |
| Figure 3 | Citation and source correctness evaluation | Simulated vignette or case evaluation | 5 | 37 | 5/37 (13.5%) |
| Figure 3 | Citation and source correctness evaluation | Workflow pilot or user study | 1 | 17 | 1/17 (5.9%) |
| Figure 3 | Citation and source correctness evaluation | Prospective clinical study | 0 | 2 | 0/2 (0.0%) |
| Figure 3 | Citation and source correctness evaluation | Real-world deployment or postdeployment monitoring | 0 | 3 | 0/3 (0.0%) |
| Figure 3 | Conflict or contradiction handling evaluation | Offline-only evaluation | 8 | 140 | 8/140 (5.7%) |
| Figure 3 | Conflict or contradiction handling evaluation | Simulated vignette or case evaluation | 3 | 37 | 3/37 (8.1%) |
| Figure 3 | Conflict or contradiction handling evaluation | Workflow pilot or user study | 1 | 17 | 1/17 (5.9%) |
| Figure 3 | Conflict or contradiction handling evaluation | Prospective clinical study | 0 | 2 | 0/2 (0.0%) |
| Figure 3 | Conflict or contradiction handling evaluation | Real-world deployment or postdeployment monitoring | 0 | 3 | 0/3 (0.0%) |
| Figure 3 | Formal safety-related evaluation | Offline-only evaluation | 39 | 140 | 39/140 (27.9%) |
| Figure 3 | Formal safety-related evaluation | Simulated vignette or case evaluation | 12 | 37 | 12/37 (32.4%) |
| Figure 3 | Formal safety-related evaluation | Workflow pilot or user study | 6 | 17 | 6/17 (35.3%) |
| Figure 3 | Formal safety-related evaluation | Prospective clinical study | 1 | 2 | 1/2 (50.0%) |
| Figure 3 | Formal safety-related evaluation | Real-world deployment or postdeployment monitoring | 1 | 3 | 1/3 (33.3%) |
| Figure 3 | Human evaluation with interrater reliability reporting | Offline-only evaluation | 23 | 140 | 23/140 (16.4%) |
| Figure 3 | Human evaluation with interrater reliability reporting | Simulated vignette or case evaluation | 9 | 37 | 9/37 (24.3%) |
| Figure 3 | Human evaluation with interrater reliability reporting | Workflow pilot or user study | 3 | 17 | 3/17 (17.6%) |
| Figure 3 | Human evaluation with interrater reliability reporting | Prospective clinical study | 0 | 2 | 0/2 (0.0%) |
| Figure 3 | Human evaluation with interrater reliability reporting | Real-world deployment or postdeployment monitoring | 0 | 3 | 0/3 (0.0%) |
| Figure 3 | LLM-as-judge evaluation with bias-control measures | Offline-only evaluation | 13 | 140 | 13/140 (9.3%) |
| Figure 3 | LLM-as-judge evaluation with bias-control measures | Simulated vignette or case evaluation | 2 | 37 | 2/37 (5.4%) |
| Figure 3 | LLM-as-judge evaluation with bias-control measures | Workflow pilot or user study | 2 | 17 | 2/17 (11.8%) |
| Figure 3 | LLM-as-judge evaluation with bias-control measures | Prospective clinical study | 0 | 2 | 0/2 (0.0%) |
| Figure 3 | LLM-as-judge evaluation with bias-control measures | Real-world deployment or postdeployment monitoring | 0 | 3 | 0/3 (0.0%) |
| Figure 3 | GraphRAG construction evaluation | Offline-only evaluation | 6 | 140 | 6/140 (4.3%) |
| Figure 3 | GraphRAG construction evaluation | Simulated vignette or case evaluation | 0 | 37 | 0/37 (0.0%) |
| Figure 3 | GraphRAG construction evaluation | Workflow pilot or user study | 0 | 17 | 0/17 (0.0%) |
| Figure 3 | GraphRAG construction evaluation | Prospective clinical study | 0 | 2 | 0/2 (0.0%) |
| Figure 3 | GraphRAG construction evaluation | Real-world deployment or postdeployment monitoring | 0 | 3 | 0/3 (0.0%) |
| Figure 3 | GraphRAG intermediate-artifact evaluation | Offline-only evaluation | 10 | 140 | 10/140 (7.1%) |
| Figure 3 | GraphRAG intermediate-artifact evaluation | Simulated vignette or case evaluation | 4 | 37 | 4/37 (10.8%) |
| Figure 3 | GraphRAG intermediate-artifact evaluation | Workflow pilot or user study | 1 | 17 | 1/17 (5.9%) |
| Figure 3 | GraphRAG intermediate-artifact evaluation | Prospective clinical study | 0 | 2 | 0/2 (0.0%) |
| Figure 3 | GraphRAG intermediate-artifact evaluation | Real-world deployment or postdeployment monitoring | 0 | 3 | 0/3 (0.0%) |

Note. Evaluation-setting categories and evaluation domains were nonmutually exclusive. For each cell in Figure 3, the numerator is the number of studies reporting the evaluation domain within the specified evaluation-setting category, and the denominator is the total number of studies in that evaluation-setting category.
