# Supplementary material for: Evaluation Methods for Inference-Time Retrieval-Augmented and Graph Retrieval-Augmented Large Language Models in Health Care: Scoping Review
Source: J Med Internet Res. 2026 Aug 3;28:e90046. doi: 10.2196/90046 (PMC13432247; doi:10.2196/90046)
Supplement: Checklist 1 [file jmir-v28-e90046-s003.docx]

**Checklist 1: Preferred Reporting Items for Systematic Reviews and Meta-Analyses Extension for Scoping Reviews (PRISMA-ScR) Checklist**

| **Section** | **Item** | **No.** | **PRISMA-ScR checklist item** | **Reported on page #** |
| --- | --- | --- | --- | --- |
| TITLE | Title | 1 | Identify the report as a scoping review. | 1 |
| ABSTRACT | Structured summary | 2 | Provide a structured summary that includes, as applicable: background, objectives, eligibility criteria, sources of evidence, charting methods, results, and conclusions that relate to the review questions and objectives. | 1-2 |
| INTRODUCTION | Rationale | 3 | Describe the rationale for the review in the context of what is already known. Explain why the review questions/objectives lend themselves to a scoping review approach. | 2-3 |
| INTRODUCTION | Objectives | 4 | Provide an explicit statement of the questions and objectives being addressed with reference to their key elements or other relevant key elements used to conceptualize the review questions and/or objectives. | 3 |
| METHODS | Protocol and registration | 5 | Indicate whether a review protocol exists; state if and where it can be accessed; and provide registration information, including the registration number, if available. | 3 |
| METHODS | Eligibility criteria | 6 | Specify characteristics of the sources of evidence used as eligibility criteria and provide a rationale. | 3-4 |
| METHODS | Information sources* | 7 | Describe all information sources in the search, as well as the date the most recent search was executed. | 4; Multimedia Appendix 1 |
| METHODS | Search | 8 | Present the full electronic search strategy for at least one database, including any limits used, such that it could be repeated. | 4; Multimedia Appendix 1 |
| METHODS | Selection of sources of evidence† | 9 | State the process for selecting sources of evidence included in the scoping review. | 4; Figure 1 (p. 8) |
| METHODS | Data charting process‡ | 10 | Describe the methods of charting data from the included sources of evidence, including whether charting was done independently or in duplicate and any processes for obtaining and confirming data from investigators. | 4 |
| METHODS | Data items | 11 | List and define all variables for which data were sought and any assumptions and simplifications made. | 5-7; Table 1; Multimedia Appendix 2 |
| METHODS | Critical appraisal of individual sources of evidence§ | 12 | If done, provide a rationale for conducting a critical appraisal of included sources of evidence; describe the methods used and how this information was used in any data synthesis. | Not applicable; no formal critical appraisal or risk-of-bias assessment conducted (pp. 6-7) |
| METHODS | Synthesis of results | 13 | Describe the methods of handling and summarizing the data that were charted. | 7 |
| RESULTS | Selection of sources of evidence | 14 | Give numbers of sources of evidence screened, assessed for eligibility, and included in the review, with reasons for exclusions at each stage, ideally using a flow diagram. | 7-8; Figure 1 |
| RESULTS | Characteristics of sources of evidence | 15 | For each source of evidence, present characteristics for which data were charted and provide the citations. | 8-10; Table 2; Figure 2; Table S1 in Multimedia Appendix 2 |
| RESULTS | Critical appraisal within sources of evidence | 16 | If done, present data on critical appraisal of included sources of evidence. | Not applicable; no critical appraisal conducted (pp. 6-7) |
| RESULTS | Results of individual sources of evidence | 17 | For each included source of evidence, present the relevant data that were charted that relate to the review questions and objectives. | 8-13; Tables 2-4; Figures 2-3; Tables S1-S4 in Multimedia Appendix 2 |
| RESULTS | Synthesis of results | 18 | Summarize and/or present the charting results as they relate to the review questions and objectives. | 8-14; Tables 2-5; Figures 2-3 |
| DISCUSSION | Summary of evidence | 19 | Summarize the main results, link to the review questions and objectives, and consider the relevance to key groups. | 14-16 |
| DISCUSSION | Limitations | 20 | Discuss the limitations of the scoping review process. | 16 |
| DISCUSSION | Conclusions | 21 | Provide a general interpretation of the results with respect to the review questions and objectives, as well as potential implications and/or next steps. | 16 |
| FUNDING | Funding | 22 | Describe sources of funding for the included sources of evidence, as well as sources of funding for the scoping review. Describe the role of the funders of the scoping review. | 16 (review funding and funder role); funding of included sources not charted |

JBI = Joanna Briggs Institute; PRISMA-ScR = Preferred Reporting Items for Systematic Reviews and Meta-Analyses Extension for Scoping Reviews.

* Where sources of evidence are compiled from, such as bibliographic databases, social media platforms, and websites.

† A more inclusive/heterogeneous term used to account for the different types of evidence or data sources that may be eligible in a scoping review as opposed to only studies. This is not to be confused with information sources.

‡ The frameworks by Arksey and O’Malley, Levac and colleagues, and the JBI guidance refer to the process of data extraction in a scoping review as data charting.

§ The process of systematically examining research evidence to assess its validity, results, and relevance before using it to inform a decision. This term is used here instead of “risk of bias” to accommodate the broader range of evidence types included in scoping reviews.

From: Tricco AC, Lillie E, Zarin W, O’Brien KK, Colquhoun H, Levac D, et al. PRISMA Extension for Scoping Reviews (PRISMA-ScR): Checklist and Explanation. Ann Intern Med. 2018;169:467-473. doi:10.7326/M18-0850.
