# Supplementary material for: Evaluation Methods for Inference-Time Retrieval-Augmented and Graph Retrieval-Augmented Large Language Models in Health Care: Scoping Review
Source: J Med Internet Res. 2026 Aug 3;28:e90046. doi: 10.2196/90046 (PMC13432247; doi:10.2196/90046)
Supplement: Checklist 2 [file jmir-v28-e90046-s004.docx]

**Checklist 2: PRISMA-S Checklist**

Reference. PRISMA-S contains 16 reporting items across information sources and methods, search strategies, peer review, and managing records. The checklist below maps the current typeset manuscript and appendix materials to those items.

| **Section/topic** | **Item** | **Checklist item** | **PRISMA-S description** | **Location(s) reported** | **Notes for this review** |
| --- | --- | --- | --- | --- | --- |
| Information sources and methods | 1 | Database name | Name each individual database searched, stating the platform for each. | Manuscript p. 4; Multimedia Appendix 1 | PubMed (MEDLINE), Web of Science Core Collection, IEEE Xplore, ACM Digital Library, arXiv, and medRxiv are named explicitly. |
| Information sources and methods | 2 | Multi-database searching | If databases were searched simultaneously on a single platform, state the name of the platform, listing all of the databases searched. | Not applicable | No simultaneous multi-database platform search was used; databases and preprint platforms were searched separately. |
| Information sources and methods | 3 | Study registries | List any study registries searched. | Not applicable; no study registries searched | No study registries were searched. |
| Information sources and methods | 4 | Online resources and browsing | Describe any online or print source purposefully searched or browsed (eg, websites, conference proceedings, tables of contents), and how this was done. | Not applicable; no structured website handsearching performed | No structured website browsing or handsearching was performed as a formal search method. |
| Information sources and methods | 5 | Citation searching | Indicate whether cited references or citing references were examined, and describe any methods used for locating cited/citing references. | Manuscript p. 4 | Backward and forward citation tracking was performed for all included studies using the same eligibility criteria. |
| Information sources and methods | 6 | Contacts | Indicate whether additional studies or data were sought by contacting authors, experts, manufacturers, or others. | Not applicable; no contact-based supplementary identification performed | No contact-based supplementary identification method was undertaken. |
| Information sources and methods | 7 | Other methods | Describe any additional information sources or search methods used. | Manuscript p. 4 | Database searching, preprint-platform searching, and backward and forward citation tracking were used; no additional formal search methods were undertaken. |
| Search strategies | 8 | Full search strategies | Include the search strategies for each database and information source, copied and pasted exactly as run. | Multimedia Appendix 1 | Full search strategies are reported for PubMed, Web of Science Core Collection, IEEE Xplore, ACM Digital Library, arXiv, and medRxiv. |
| Search strategies | 9 | Limits and restrictions | Specify that no limits were used, or describe any limits or restrictions applied to a search and provide justification for their use. | Manuscript p. 4; Multimedia Appendix 1 | Searches were limited to English-language records published or posted from January 1, 2024, through May 14, 2026; the rationale is provided in the manuscript. |
| Search strategies | 10 | Search filters | Indicate whether published search filters were used (as originally designed or modified), and if so, cite the filter(s) used. | Not applicable; no published search filter used | No published search filter was adopted or adapted. |
| Search strategies | 11 | Prior work | Indicate when search strategies from other literature reviews were adapted or reused for a substantive part or all of the search, citing the previous review(s). | Not applicable; no prior review search strategy reused | The search strategy was developed iteratively by the review team and was not adapted from a prior review. |
| Search strategies | 12 | Updates | Report the methods used to update the search(es) (eg, rerunning searches, email alerts). | Not applicable; searches were not updated after the final searches conducted on May 14, 2026 | The final searches were conducted on May 14, 2026. No subsequent update search or automated alert was used. |
| Search strategies | 13 | Dates of searches | For each search strategy, provide the date when the last search occurred. | Manuscript p. 4; Multimedia Appendix 1 | The last search date was May 14, 2026, for each information source. |
| Peer review | 14 | Peer review | Describe any search peer review process. | Not performed; no formal search peer review conducted | The search strategy was not formally peer-reviewed before execution (eg, no PRESS or information-specialist review). |
| Managing records | 15 | Total records | Document the total number of records identified from each database and other information source. | Manuscript p. 8, Figure 1 | The PRISMA flow diagram reports the number of records identified from each database and preprint platform before deduplication. |
| Managing records | 16 | Deduplication | Describe the processes and any software used to deduplicate records from multiple database searches and other information sources. | Manuscript p. 4; Figure 1 (p. 8) | Records were deduplicated in EndNote before screening; the number of duplicate records removed is shown in Figure 1. |
